# A systematic approach to the modelling and comparison of the geometries of spherical electrodes in inertial electrostatic confinement fusion devices

**DOI:** 10.1038/s41598-024-52173-6

**Published:** 2024-01-27

**Authors:** Jan-Philipp Wulfkühler, Hai-Dang Nguyen, Leo Peiffer, Martin Tajmar

**Affiliations:** Institute of Aerospace Engineering, Technische Universität Dresden, 01307 Dresden, Germany

**Keywords:** Experimental nuclear physics, Nuclear fusion and fission

## Abstract

Inertial electrostatic confinement fusion (IECF) devices often use two concentric spherical electrodes to converge ions in a plasma electrostatically. Using a highly transparent inner cathode, the ions can move through the cathode and collide at the center to undergo fusion reactions. This is a simple method to build a neutron source. Past research has focused chiefly on cathode “grids” manufactured by joining metal wire loops or disc-shaped elements via spot welding. There are two common geometries: “Globe” grids with a distinct latitude-longitude structure and “symmetric” grids with even-sized triangular-shaped apertures. Recent advances in additive manufacturing have opened the way to manufacturing a third class of grids in which the apertures are evenly distributed over the grid surface and have either circular or polygonal shaped apertures - here called “regular” grids. These three types are analyzed and compared based on a set of metrics, including transparency, homogeneity of aperture size, and the regularity of aperture distribution. It is shown that every type of grid comes with different advantages and disadvantages. The analysis focuses on grid geometries with 6 to 120 apertures.

## Introduction

**Figure 1:**
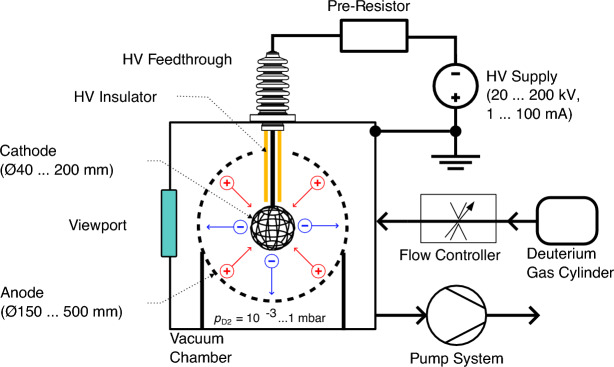
Simple schematic of IECF device with spherical cathode and concentric anode based on gas discharge with deuterium gas as fusion fuel. Typical electrode dimensions are given in brackets. Ions of the gas discharge are electrostatically confined between the electrodes, whereas electrons are unconfined. Fusion reactions originate mostly from beam-background and beam-surface reactions (see Chapter 8 in Ref.^8^ for a full discussion).

Inertial electrostatic confinement fusion (IECF) uses electrostatic potential well structures to confine and accelerate ions to achieve nuclear fusion conditions. Typically, IECF devices rely on a spherical geometry with a central cathode and an outer anode. There exist many different concepts for IECF, which can be broadly distinguished into two main classes based on their type of cathode: The first class is comprised of IECF devices with a virtual cathode, in which a cloud of electrons attracts and confines ions (such as in the Polywell^1^ or modified Penning trap^2,3^). The second class evolves around devices with a physical electrode acting as the cathode at the center of the device (see Fig. 1). Most IECF devices employ only a single cathode “grid” but there exist designs with multi-grids^4^ and even concepts with continuous electromagnetic focus grids^5^. This paper is focused on design aspects of the cathode of single-grid IECF devices, representing the simplest design. Although they suffer from ion losses due to collisions with a grid, which is the main factor that prevents these devices’ upscaling, they have many near-term applications as a neutron source. The overall record neutron production rate of 3.8$\cdot10^{8}\,\text{s}^{-1}$ for steady state operation (discharge at 200 kV and 100 mA)^6^ and 5$\cdot 10^{9}\,\text{s}^{-1}$ for pulsed operation (discharge at 115 kV and 2 A with pulse length of 0.5 ms at 10 Hz)^7^ with deuterium equals a total fusion power of just 0.44 mW and 5.8 mW respectively.

**Figure 2:**
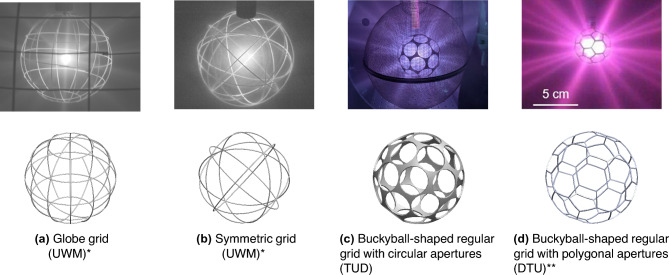
Different types of cathode geometries during deuterium glow discharge operation in IECF devices by various research groups (UWM - University of Wisconsin-Madison, TUD - Dresden University of Technology, DTU - Technical University of Denmark). The two grids on the right with a “buckyball”-shaped geometry belong to a novel class of spherical IECF grids (regular grids) which are analyzed in this paper. *reprinted from Ref.^17^ by permission of the publisher (Taylor & Francis Ltd, http://www.tandfonline.com ). **Reprinted from Ref.^12^, with the permission of AIP Publishing.

High-power gridded IECF devices have mainly relied on two types of grid structures: Grids made from individual loops that are arranged in a latitude-longitude structure (see Fig. 2a) and grids in which same-sized loops form a more symmetric structure whose apertures form similar-sized triangles (see Fig. 2b). In this paper, these grids will be referred to as “globe grids” and “symmetric grids” respectively, terms which have been widely used in the IECF literature^8^. Both types of grids have a high geometric transparency and the property, that for each aperture exists another opposite (antipodal) aperture to ensure a free circulating flow of ions within the electrostatic potential well. This is an essential prerequisite for IECF.

Recently, the advances in commercially available additive manufacturing - in particular the selective laser melting technology - have sparked interest in testing spherical grids with 32 apertures, which are distributed similarly to the faces of a truncated icosahedron - often known from the C60 “Buckyball” fullerene. Tests with circular shaped apertures (see Fig. 2c) were conducted^9–11^ as well as with polygonal shaped apertures (see Fig. 2d)^12^. However, the case of 32 apertures, which are very evenly distributed over a spherical surface, represents only one special configuration. Indeed, additional tests with grids based on the cube (6 apertures) and the dodecahedron (12 apertures) have been presented in Ref.^13^. So far, these tests have only been conducted by a few research institutes and have been limited to a certain range of operating conditions of the IECF devices (e.g. plasma discharge power level). As of now, it is unclear, if their geometries can provide inherent advantages over the “classic” globe grids or symmetric grids in terms of enhancing the fusion rate of IECF devices.

The motivation for this paper is, therefore, threefold:

1. Present a thorough description of the geometry of the different types of spherical IECF cathode geometries
2. Introduce a new class of IECF grids: “regular”-shaped grids
3. Compare globe grids, symmetric grids and regular-shaped grids based on a set of metrics.

### A new type of cathode geometry: regular-shaped grids

As mentioned above, a prerequisite for spherical IECF cathodes is that each aperture is faced by another aperture on the opposite side (antipodal symmetry) so that ions can freely flow along radial lines through the cathode. Therefore, every even number of apertures evenly distributed over the surface might be the basis for an IECF cathode grid. Here, these grids will be referred to as “regular”(-shaped) grids, based on the even or regular distribution of the apertures. This terminology is not meant to be a new standard; other better-suited terms might be suggested in future works. Two sub-types are distinguished: Regular grids with circular apertures and regular grids with polygonal apertures. The distribution of these apertures might at first seem to be random but is instead a product of numerical optimization algorithms based on the mathematical problem of the optimum packing of circles on the surface of the sphere with the aim of maximizing the circle diameter. As seen from Fig. 2c, the circular apertures lead to a comparatively low transparency. This paper will show that several more potential candidates might be of interest for the application in IECF devices ($N=$ 44, 58, 78, 96, 110, 120). However, except for the case of 6 and 12 apertures, no analytical solution is known for an optimum distribution, and instead, a numerical iterative optimization approach is required. (In fact, the truncated icosahedron does not represent the best solution^14^, but the deviation is so small that it is not relevant for the design of IECF grids.) This work uses pre-calculated distributions from the mathematical field of circle packaging on a sphere (published primarily by Sloane et al.^14,15^) and also presents a simple algorithm (based on work by Gautam and Vaintrob^16^) which can calculate these distributions quickly and with a reasonable precision to analyze the geometries further and generate CAD geometries for rapid manufacturing. The aim is not to obtain exact optimum solutions - there are many configurations for which the optimum configuration has not been proven yet - but to quickly generate the geometry with the required antipodal symmetry. The authors have briefly discussed parts of this optimization procedure in Ref.^9^.

### Metrics for comparison of grid geometries

Besides the geometric description of the novel grids, the second intent of this work is the comparison between the different grid types. To achieve a fair comparison between them requires the introduction of several figures of merit. Four figures of merit were identified:

1. Geometric transparency,
2. Homogeneity of aperture size,
3. Circular transparency,
4. Homogeneity of the distribution of the apertures over the spherical grid structure.

A detailed explanation is given in later chapters. So far, of these four metrics, only the transparency and the homogeneity of the size of apertures have been researched. The other two represent geometric metrics that are not necessarily linked to the performance of real IECF devices. This has to be analyzed in future experiments.

### Structure of the paper

The following section starts with a review of traditional cathode geometries for spherical gridded IECF devices and then briefly summarizes recent developments in the application of the buckyball-shaped geometry and similar geometries in IECF devices, which motivated the research of this paper. Also, requirements for the cathode grid design are summarized from the literature. The chapter closes with a critical discussion on the question if regular shaped grids can indeed improve the performance of IECF devices. Chapter Systematic description of spherical grid geometries describes the systematic approach to the modelling of the geometry of the different types of grids. This is followed by an overview of the four metrics used to systematically compare the grid types with each other in Chapter Metrics for the comparison of different types of spherical grids. The detailed analysis of the different grid types is presented in Chapter Parameters of globe grids (globe grids), Chapter Parameters of symmetric grids (symmetric grids) and Chapter Modelling of regular-shaped grid geometries (regular grids with circular and polygonal apertures). Chapter Modelling of regular-shaped grid geometries also contains an overview of procedurally generated geometries of regular-shaped grids. All grids are compared in Chapter Comparison of parameters of different grid types. The paper concludes with a summary and outlook. A detailed description of the underlying equations for the geometries and metrics is presented in the Appendix to keep the main text concise.

## Overview of grid designs for IECF devices

### Traditional cathode geometries

Early IECF designs from the 1970s relied on cathodes made from very fine meshes. e.g. Dolan et al.^18^ employed meshes with spacings in the range of roughly 0.3 to 4 mm. Similarly, Black and Robinson^19^ used meshes with 0.025 mm wire diameter and a spacing of 0.31 mm wrapped over a spherical support structure. However, these experiments aimed to build devices with extremely high spherical symmetry to confine fusion plasmas in multipotential well structures. These tests proved to be inconclusive.

With a shift of focus to near-term applications of the IECF concept for neutron sources, Miley et al.^20–22^ introduced grids with larger apertures for beam-background dominated IECF devices, which rely on Paschen discharge plasmas or simple thermionic filaments to support the discharge. During the experiments, the “star”-mode discharge was discovered, in which ion microchannels appear at the center axis of the apertures. Because these microchannels show a higher ionization degree than the surrounding plasma, the losses due to grid collisions are significantly minimized. This became a preferred discharge mechanism due to its simplicity.

As already discussed in the introduction, two different types of cathode grids became the standard for spherical gridded IECF devices: The globe grids and symmetric grids. The following list summarizes more experimental findings about these grids, which are later important to compare them to the novel grid designs:

- Murali et al.^23^ conducted a detailed study on the influence of the grid structure on the fusion rate. By sequentially increasing the number of wire loops forming the cathode from a single loop to a grid comprised of 12 longitudinal and 5 latitude wires with 36 apertures, it was shown that the neutron production increased at first but then saturated as soon as the grid achieved a certain complexity.
- In 2017 Michalak et al.^24^ set a D-D fusion record of $2.5\cdot 10^8$ n/s (at 150 kV, 100 mA and 1 mTorr) with a cathode made from 9 latitude and 16 longitude rings and approximately 160 apertures.
- In 2018 this record was surpassed by Fancher with $3.8\cdot 10^8$ n/s (200 kV, 100 mA and 1 mTorr). Fancher upgraded the cathode used by Michalak with more longitude rings. The final grid consisted of 9 latitude and 32 longitude rings and approx. 640 apertures. The addition of 16 longitude grids contributed to stabilizing the plasma discharge at voltages above 180 kV. Although adding more segments to the grid reduced the transparency, it also helped to reduce the thermal load on the grid locally.
- In 2021 Bakr et al.^25^ demonstrated a neutron production rate of $9.2\cdot 10^7$ n/s at 80 kV and 80 mA at the Kyoto University. The symmetrical grid made from 6 molybdenum discs featured 24 apertures.
- Wehmeyer^17^ compared the performance of a symmetrical style grid (9 rings, 48 apertures) to a globe grid (12 lat, 5 long, 48 apertures) and the neutron production rates showed no significant difference.

### Requirements for spherical grids

The most important requirements (RQ) for spherical cathode electrodes in IECF devices are listed below (for a more in-depth discussion, see Chapters 5 and 6 in Ref.^8^). Note that these requirements have been developed or discovered for globe grids and symmetric grids but are likely transferable to the class of regular-shaped grids.

- RQ1 High geometric transparency: To minimize the loss of ions to the surface of the cathode, it should have a high transparency. An often cited equation to estimate the maximum number of passes $\#p$ an ion can perform that is based solely on the transparency $\eta$ is Ref.^26^:
  1 $$\begin{aligned} \#p = \frac{\eta }{1-\eta ^2}, \end{aligned}$$
- RQ2 High effective transparency by antipodal symmetry: The term *effective* transparency was coined by Miley et al.^20^ and takes the requirement for the antipodal symmetry of approximately similar-sized apertures into account.
- RQ3 High symmetry of apertures: To obtain a spherical homogeneous and symmetric discharge, the apertures should have similar sizes and be evenly distributed. An uneven grid structure can lead to non-symmetric discharges. A special case is the jet mode, in which the plasma discharge is characterized by a single jet (predominantly composed of electrons), which emerges from the cathode (usually from the largest aperture)^27^. Although this discharge type can lead to high neutron production rates, it is not always preferred.
- RQ4 High resistance against sputtering and temperature compatibility: Different materials have been employed as cathode materials. The selection varies from stainless steel with a relatively low melting point to refractory metals like rhenium and tungsten. The latter two also have a good resistance against sputtering, which considerably reduces impurities in the plasma, unwanted coating of insulators, premature breakdown on roughened surfaces and generally prolongs the lifetime of the grids in the harsh environment. Even graphite has been employed recently^28^.
- RQ5 The apertures should not be too small to allow for the development of microchannel discharges, which improve the fusion rate^20,21^.
- RQ6 Manufacturing: The electrodes need to be manufacturable with a reasonable effort. Typical methods involve spot welding of wires (which can be simplified with a jig)^4,7^ or the joining of precisely cut metal sheets^29^.
- RQ7 Optimum diameter of cathode: The diameter of the cathode depends on several factors like the size of the IECF device, manufacturing costs and the anode’s size. In general, larger cathode diameters are preferred for high-pressure devices which rely on beam-background fusion. For beam-beam fusion, smaller cathodes are preferred.^30^ For the discussion of the geometry the diameter is not of interest and a unit sphere is assumed as an underlying structure that can be scaled as desired.

### Buckyball-shaped grids and similar-shaped grids in IECF devices

In the following section, a brief review of the use of Buckyball-shaped grids and similar geometries is given - which represent the limited number of configurations tested of the regular grid class. In 2010, Sedwick et al.^31^ proposed an IECF concept based on multiple nested buckyball-shaped grids with spherical apertures made from a magnetic material to divert the ions and electrons from impacting the grids and also to minimize particle loss to the anode. The grids would have to be manufactured from magnetic rare earth materials, a technique that is still not available. This theoretical concept was further developed by Chap in his PhD thesis in 2017^5^ with one “continuous” grid instead of multiple grids. This grid, which features polygonal apertures, is very thick (the thickness almost equals the radius).

**Figure 3:**
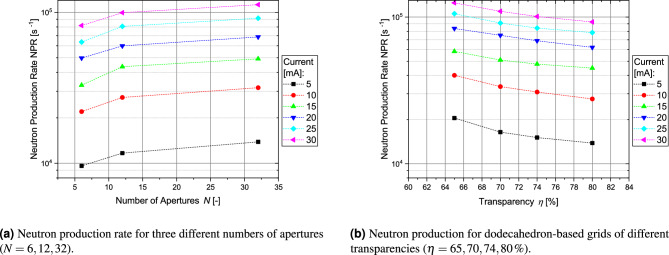
Neutron production rates measured with cube-, dodecahedron- and buckyball-based grid geometries with circular apertures^13^.

The authors conducted the first experiments with buckyball-shaped grids with circular apertures in 2016^9^. Several grids were manufactured with selective laser melting (SLM) made from stainless steel and Ti-6Al-4V with diameters of 40 and 150 mm. Discharge tests with argon showed that typical glow discharge characteristics like “star”-mode and “jet”-mode could also be observed with this type of grid. These grids featured circular apertures, a decision that was driven by two factors: First, the influence of circular-shaped apertures should be explored, and second, at that time, it was not possible to have these grids with polygonal-shaped apertures since the thinner structures could not be manufactured with the SLM technology available at that time. For subsequent analysis, these grids were sent to Bowden-Reid et al.^10^ for fusion tests with deuterium. Notably, Bowden-Reid et al. were able to show that a significant portion of the fusion reactions occurs at the surface of grids (in a subsequent publication by the research group^28^ it was theorized that surface fusion seems to be the predominant mechanism in lower power discharges and that high power discharges will predominantly show beam-background fusion as described in Ref.^32^). Bakr et al.^11^ measured the neutron production rate from two buckyball-shaped grids with a diameter of 50 mm at voltages of up to 80 kV and 80 mA. One grid was made from stainless steel, the other from titanium (Ti-6Al-4V). The fusion rate of the titanium grid proved to be 1.36 to 1.64 times higher compared to the stainless steel grid. This also supports Khachan’s claims that the fusion rate depends significantly on surface fusion. This hypothesis was further reinforced by additional tests by Bakr et al.^13^ with different stainless steel grids with 6, 12 and 32 circular apertures. However, problems with the surface quality and the grid material limited the maximum discharge voltage to 30 kV and 30 mA in the tests. The measurements indicated a potential increase in the neutron production rate for a higher number of apertures (see Fig. 3a). Unexpectedly, the neutron production rate of grids with 12 apertures increased for lower transparencies (see Fig. 3b). This comparison was not conducted for the grids with 6 and 32 apertures.

In 2020, Rasmussen et al.^12^ published a study that compared the performance of several highly transparent spherical grids and cylindrical grids made from titanium and tungsten. The grid specimen included designs with the buckyball-shaped geometry but with polygonal apertures instead of circular ones. They referred to this as “soccerball” design. Grids with diameters of 40, 80, 120 and 160 mm and wire diameters of 1 mm were manufactured. A comparison between these grids and similarly sized globe grids with 16 apertures demonstrated an increase of the neutron production rate of the regular-shaped grid by 25 %. Rasmussen et al. also discussed the possibility of using geometries with a higher number of apertures and a higher degree of symmetry but did not discuss specific geometries. They also addressed the issues of uncertainty for an increase of the fusion rate with a higher number of apertures (referring to the work by Murali^23^) and the problems that might arise if the apertures are reduced beyond a certain critical limit in which the star-mode discharge might be inhibited.

At the University of Stuttgart, grids were made with additive manufacturing for a non-fusion propulsion concept based on IEC technology but with slightly different geometries (based on a modified truncated cuboctahedron pattern) and non-fusion propellants^33^.

It has to be noted that selective laser melting is currently readily available for stainless steel as well as Ti-6Al-4V. But materials like molybdenum, which is often used in symmetric grids made from laser-cut disk-elements^34^, as well as Rhenium and Tungsten, which are often used in wires (especially as alloy W-25 Re^32^) are not widely available. In theory, 5-axis CNC machining could also manufacture such geometries from a solid block or prepared spherical shells, but it would be very difficult.

### Critical questions regarding the regular-shaped grid designs

From the review of both traditional grids and the first tests of the regular grids, the following questions were derived:

- Assuming similar parameters (e.g. the diameter of the cathode, material, number of apertures, and discharge conditions): How do grids with a buckyball structure compare to classic globe grids or symmetric grids? Although not conducted with a similar number of apertures, the study by Rasmussen et al.^12^ showed a gain of 25 % for the neutron production rate of grids with a similar diameter and same-sized bridges from globe grids (16 apertures) and a buckyball-shaped grid with polygonal apertures.
- How do regular grids with circular apertures compare with grids of polygonal apertures? This has yet to be tested.
- How do grids with different numbers of apertures perform? Only grids with 6, 12 and 32 circular apertures^11,13^ and grids with 32 polygonal apertures^12^ have been tested. It will be shown in this paper that there exist multiple other promising configurations with a higher number of apertures.
- The tests by Bakr et al.^11,13^ indicate that surface fusion plays a significant role and under certain conditions grids with lower transparency show a better performance (see also Fig. 3b). It is unclear how this trend evolves for transparencies below 65 % and above 84 %. Bowden-Reid et al.^28^ showed that in low-power IECF devices (below 1 kW) surface fusion effects play a predominant role (up to 80 %), but at higher power levels beam-background fusion reactions dominate^32^.

Also, the review of the state-of-the-art grid designs raised the following critical points:

- The manufacturing of these grids can be extremely cost-intensive since the price scales approximately proportionally to the volume. In addition, refractory metals like molybdenum, rhenium and tungsten (and their alloys) are still not readily available. As of now, with selective laser melting special attention has to be paid to avoid warping of the geometry during printing, avoid rough surfaces and clean removal of support structures during the printing process. These drawbacks might limit the use of the novel grids for some time in high-power IECF devices until the manufacturing technologies have matured sufficiently.
- Especially for grids with circular-shaped apertures, it is still unclear if the circular apertures with a more homogeneous field distribution can benefit the IECF operating process. The reduced transparency might lead to increased ion losses due to collisions and a reduction in the effectiveness of the star-mode, which requires apertures of a specific size. Debye-shielding might cause this as the aperture size approaches the size of the Debye length in the plasma, and the particle beams no longer “see” the potential drop at the apertures.
- As it will be shown in Sect. Analysis of results, regular grids with circular-shaped apertures with $$N = 6,12,32,44,58,78,96,110$$ and 120 apertures show the highest transparency. However, as Murali et al. demonstrated in their experiments, the fusion rate seemed to saturate above a certain number of apertures (around $$N=48)$$. Therefore, half of the calculated configurations might not deliver a significant gain. Tests by Bakr also indicated a saturation for a higher number of apertures (see Fig. 3a). By doubling the number of apertures from 6 to 12, the neutron production rate increased by nearly 25 % on average. However, the more than 5 times higher number of apertures $$N=32$$ increased the neutron production rate by only 43 %.
- It also needs to be clarified if the more even distribution of apertures can lead to significant gains as Radel^7^ could not detect significant differences in the fusion rates of globe grids and symmetric grids.
- Fancher^6^ made several considerations based on his record neutron-producing test with a lat.-long. shaped wire grid, which was described in the previous section. As a result of discharge instabilities caused by sharp points of the cathode grid, he proposed the development of cathode grids made from additive manufacturing of tungsten to reduce local electric field spikes and improve the uniformity of the apertures. Fancher also observed that at high power levels, the alignment of the cathode and the anode with respect to their apertures is critical.

Currently, it is not possible to estimate if regular-shaped grids possess an advantage over globe grids or symmetric grids in terms of the specific fusion rate - the fusion rate per input power. Only an extensive test campaign with an IECF device that tests all grid types under similar conditions under a wide range of parameters (discharge voltage, current and pressure) can answer this question. Table 1 compares the advantages and disadvantages of the different grid types.

**Table 1:** Summary of advantages and disadvantages of different grid types.

Grid type	Advantages	Disadvantages
Globe grid	$\bullet$ Simple to manufacture by spot welding of wire loops $\bullet$ Spot welding allows easy and cost-effective manufacturing of larger cathode and anode grids $\bullet$ High flexibility with regards to complexity by simple addition of loops	$\bullet$ Irregular distribution of uneven shaped apertures $\bullet$ Usually sharp edges and joints if manufactured by spot welding
Symmetric grid	$\bullet$ Similar shaped apertures	$\bullet$ Limited flexibility with regards to number of apertures
Regular grid - circular apertures	$\bullet$ Apertures of same size with regular distribution $\bullet$ Larger bridge/joint size can improve the additive manufacturing process compared with polygonal apertures	$\bullet$ Can only be manufactured by additive manufacturing $\bullet$ Low transparency
Regular structure - polygonal apertures	$\bullet$ High transparency $\bullet$ Excellent distribution of apertures	$\bullet$ With current additive manufacturing technology the fragile small structural elements easily deform

## Systematic description of spherical grid geometries

**Figure 4:**
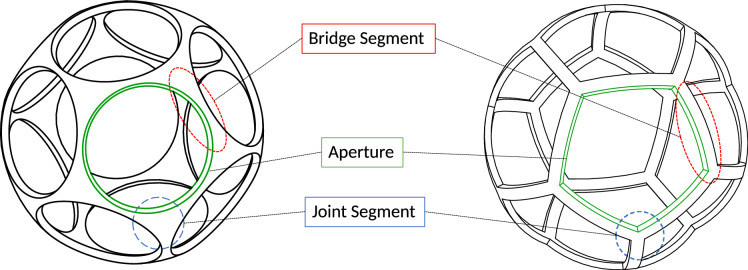
Grid segments: The elements separating two apertures are referred to as bridge segments. The joints connecting (usually three) bridge segments are referred to as joint segments.

In this chapter, a rigorous approach to the description of the geometries of the different grid types is presented. The basic shape of the spherical grids is described by a spherical shell with an outer radius $R_\text {grid}$ and a thickness $t_\text {grid}$. Individual apertures can be modeled by the Boolean difference between the spherical shell and either pyramid-shaped objects (in the case of polygonal apertures) or cones (in the case of circular apertures). The elements between the apertures will be referred to as bridge segments and the joints connecting two or more bridges will be referred to as joint segments (see Fig. 4).

**Figure 5:**
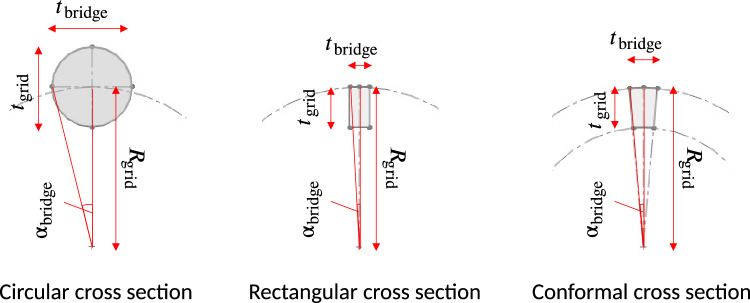
Three main types of bridge segment cross sections in IECF cathodes.

The bridges can have different cross-sections. For the present analysis, three types (see Fig. 5) can be distinguished: Circular, rectangular and conformal grid cross-sections. Circular cross-sections are typically found in grids made from wire loops or segments. In that case, the wire diameter $d_\text {wire}$ equals the bridge thickness $t_\text {bridge}$ as well as the grid thickness $t_\text {grid}$. The center point of the circular cross-section is assumed to be on the radius of the grid $R_{grid}$. The rectangular cross-section is usually encountered in grids made from disc-shaped elements. Conformal cross sections (geometrically described by an annulus sector) are difficult to manufacture and usually require additive manufacturing technologies. For most of the analysis, conformal cross sections with a constant bridge thickness $t_\text {bridge}$ are used (unless noted otherwise) because, in that case, the grid transparency is independent of the grid radius.

**Figure 6:**
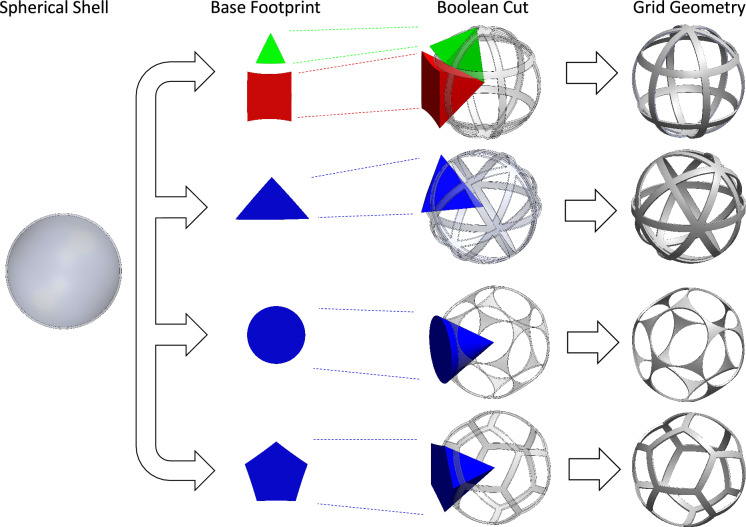
Visualisation of generation of grid geometry by cutting apertures into spherical shell.

Apertures can be modeled by the Boolean difference with irregular n-sided pyramid-shaped or cones, as shown in Fig. 6. Note that the base of these cones is made from convex surfaces. This is a direct result of the bridge segments between the apertures, which are formed by great circles. Only globe grids with latitudinal segments that are small circles also feature concave-shaped cuts.

For the present analysis, a grid radius of $R_\text {grid}=1$ is assumed to make it independent of size and units. Therefore, the outer surface of the spherical shell $S^2$ can be described as:

2 $$\begin{aligned} S^2:=\{\hat{{{\textbf {p}}}}\in {\mathbb {R}}^3: ||{{\textbf {p}}}||=1 \}, \end{aligned}$$

**Figure 7:**
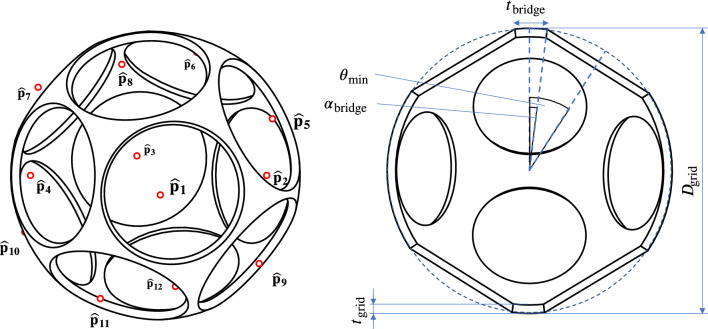
Geometric properties of grids demonstrated in the cut view of a regular-shaped grid with circular apertures based on a dodecahedron ($N = 12$). Note that the transparency of the grid was deliberately chosen to be small (large $t_\text {bridge}$) in order to improve visual clarity of the grid properties.

The number of apertures of the grid is denoted by *N*. The position of each aperture can be described by its center-point  $\hat{{{\textbf {p}}}}_i$. The center-point is projected on the outer surface of the spherical shell (see Fig. 7). Together, the *N* points create a set *P*, which can be divided into two subsets based on their antipodal symmetry:

3 $$\begin{aligned} P:=\{\hat{{{\textbf {p}}}}\}_{i=1}^{N} = \{\hat{{{\textbf {p}}}}_{i}^{+} \}_{i=1}^{N/2} \cap \{\hat{{{\textbf {p}}}}_{i}^{-} \}_{i=1}^{N/2}, \quad N=2h, \ h = 1,2,3, \dots, \end{aligned}$$

These subsets are related via the unit matrix ${{\textbf {E}}}$ as follows:

4 $$\begin{aligned} \hat{{{\textbf {p}}}}_{i}^{-} = -{{\textbf {E}}}\hat{{{\textbf {p}}}}_{i}^{+}, \end{aligned}$$

The size of the apertures is limited by its nearest neighboring aperture and the bridge element between. The half of the angular distance between the closest neighbors is donated as $\theta _\text {min}$ (see Fig. 7). Through their conformal cross-section, the bridges are associated with a half-angle $\alpha _\text {bridge}$. The thickness of a bridge element $t_\text {bridge}$ - a projection of the grid thickness onto the grid radius $R_\text {grid}$ - can therefore be expressed by:

5 $$\begin{aligned} \tan (\alpha _\text {bridge})=\frac{\frac{t_\text {bridge}}{2}}{R_\text {grid}}=\frac{t_\text {bridge}}{D_\text {grid}}, \end{aligned}$$

Throughout the following geometric analysis, the five specific grid angles of Table 2 will be used. Since the analysis will be carried out for a grid radius $R_\text {grid} \equiv 1$ and the bridge ratio and the half-angle are dimensionless, the average aperture area will be presented as a dimensionless value. However, $R_\text {grid}$ will be included in all equations to not lose the generality.

**Table 2:** Overview of grid angles for calculation.

#	$\frac{t_\text {bridge}}{D_\text {grid}}$ ratio	Grid angle $\alpha _\text {bridge}$ [^∘^]	Description
A1	0	–	Limit case of zero thickness between apertures (maximum aperture angle)
A2	$\frac{1}{50}$	1.146	Represents cathode with diameter of 50 mm and bridge thickness of 1 mm
A3	$\frac{1}{100}$	0.573	Represents cathode with diameter of 100 mm and bridge thickness of 1 mm
A4	$\frac{1}{150}$	0.382	Represents cathode with diameter of 150 mm and bridge thickness of 1 mm
A5	$\frac{0.8}{200}$	0.229	Represents cathode with diameter of 200 mm and bridge thickness of 0.8 mm (close to values in^24^)

## Metrics for the comparison of different types of spherical grids

To assess and compare the different grid types, a set of metrics is required. These metrics are derived partly from the general requirements for spherical cathode grids stated in Sect.  Requirements for spherical grids and partly from the geometric intricacies of the grids themselves. The metrics are:

1. Geometric transparency,
2. Homogeneity of aperture size,
3. Circular transparency,
4. Homogeneity of distribution of apertures over spherical grid (“Potential Energy”).

**Figure 8:**
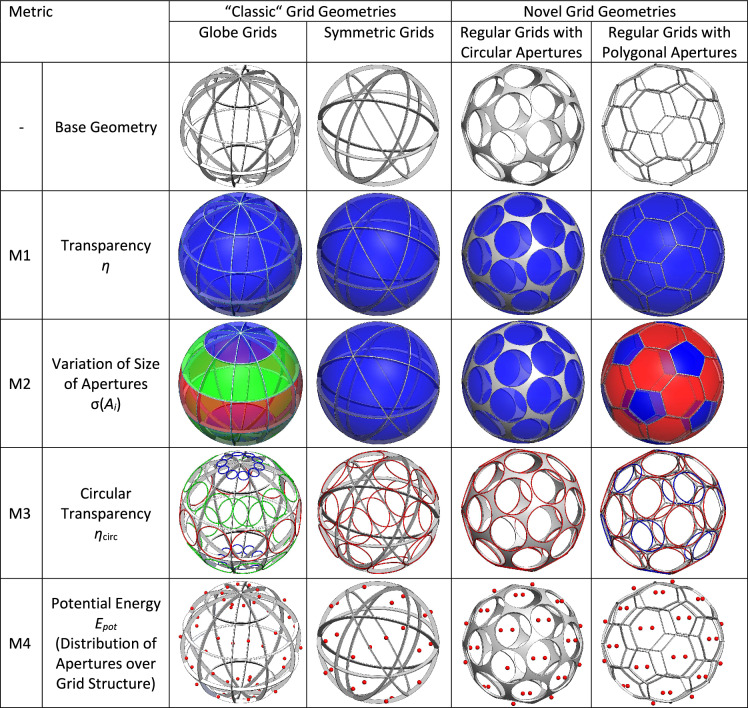
Visualisation of metrics for different grid types (globe grid with 4 latitudes and 9 longitudes, symmetric grid made from 6 segments, regular grids based on Buckyball with either 32 circular or 32 polygonal apertures).

A visualization is presented in Fig. 8. The geometric transparency $\eta$ (metric M1) is generally computed by the ratio of the sum of the individual spherical surface areas $A_{i}$ of the apertures and the total area of the underlying sphere.

6 $$\begin{aligned} \eta = \dfrac{\sum _{i=1}^{N}A_{i}}{4\pi R_\text {grid}^2}, \end{aligned}$$

As discussed in Requirement RQ1 in Sect. Requirements for spherical grids, a high geometric transparency is associated with better performance of IECF devices because the ion losses due to surface collisions are minimized. To obtain a symmetric discharge, two more conditions should be fulfilled: First, the antipodally distributed grids should have a regular or systematic (globe grid, symmetric grid) distribution, and second, the apertures should have a similar size. Therefore, the average aperture size *A* and the standard deviation of the individual aperture areas $\sigma (A_{i})$ play an important role in the assessment of the grids (metric M2).

**Figure 9:**
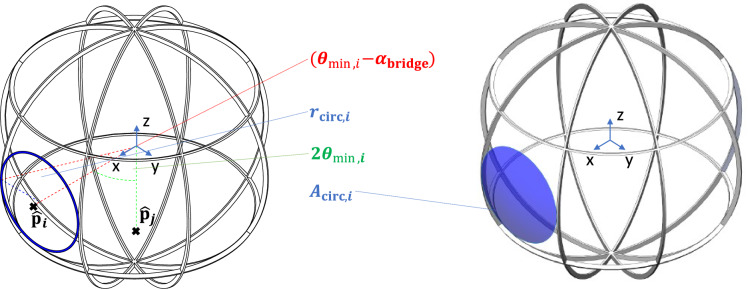
Visualization of the circular transparency with a globe grid ($N=24$).

The third metric is referred to as circular transparency. It describes the maximum transparency if the apertures have a circular shape. By comparing the geometric transparency with the circular transparency, an indicator is given, of how close the shape of the apertures is to one of circles. Circular-shaped apertures have not been experimentally proven to have an advantage over polygonal-shaped apertures with higher geometric transparency regarding neutron production rate per power. Under most conditions, the ion beams will likely fill out the complete aperture - circular or polygonal-shaped - with a fall-off due to the Debye-sheath close to the cathode surface^12,35^. However, circular apertures have a less distorted electric field than polygonal apertures. This could prove advantageous for grid configurations, especially multi-grid configurations, in which multiple concentric grids are used as electrostatic lenses to focus the ion beams (see Refs.^4,5,30^). Figure 9 presents a visual representation of the circular transparency of a single aperture. The minimum half-angle $\theta _{\text {min,}i}$ of a specific aperture together with the bridge half-angle $\alpha _\text {bridge}$, define the radius of the base of the spherical cap with surface area $A_{\text {circ,}i}$. This is expressed by the following two equations:

7 $$\begin{aligned} r_{\text {circ,}i}&= R_\text {grid} \sin {(\theta _{\text {min,}i}-\alpha _\text {bridge})}, \end{aligned}$$

8 $$\begin{aligned} A_{\text {circ,}i}&= 2\pi R_\text {grid}^2 \left( 1-\cos {(\arcsin {(\dfrac{r_{\text {circ,}i}}{R_\text {grid}})}})\right), \end{aligned}$$

The circular transparency $\eta _\text {circ}$ is then calculated as:

9 $$\begin{aligned} \eta _\text {circ} = \dfrac{\sum _{i=1}^{N}A_{\text {circ,}i}}{4\pi R_\text {grid}^2}, \end{aligned}$$

Based on the transparency $\eta$ and the circular transparency $\eta _\text {circ}$ the normalized circular transparency ${\hat{\eta }}_\text {circ}$ is introduced:

10 $$\begin{aligned} {\hat{\eta }}_\text {circ} = \dfrac{\eta _\text {circ}}{\eta }, \end{aligned}$$

The normalized circular transparency describes the average fraction of the aperture areas that are part of the spherical caps that define the circular transparency. Therefore, the regular-shaped grids with circular apertures have a normalized circular transparency of ${\hat{\eta }}_\text {circ} = 1$.

The distribution of the apertures over the spherical surface can be assessed by a fourth metric (M4) described here as potential energy $E_\text {pot}$ or sometimes also known as s-energy^36^. These terms have been borrowed from the problem of spherical packaging and are assessed in more detail in Appendix B.1 andB.2. From the location of the center points $\hat{{{\textbf {p}}}}_i$ the following equation can be deduced:

11 $$\begin{aligned} E_\text {pot}(\hat{{{\textbf {p}}}}_1, \hat{{{\textbf {p}}}}_2,... \hat{{{\textbf {p}}}}_{N}) = \sum _{i\ne j}^{N}\frac{1}{||\hat{{{\textbf {p}}}}_i - \hat{{{\textbf {p}}}}_j ||^s}, \end{aligned}$$

**Figure 10:**
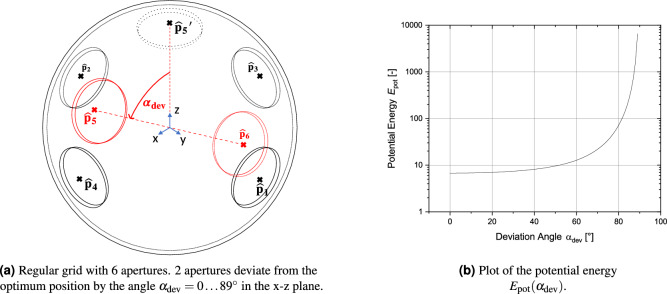
Visualization of the potential energy metric for a regular shaped grid with $N=6$ circular apertures. 2 apertures ($\hat{{{\textbf {p}}}}_5$ and $\hat{{{\textbf {p}}}}_6$) deviate from their optimal position by the angle in the x-z plane. Note that there is not an ideal distribution for every *N*.

A lower potential energy means that the mean distance between the points is maximized; therefore, the distribution of the apertures should be more even. This is shown in Fig. 10.

## Parameters of globe grids

### Overview of geometrical properties

**Figure 11:**
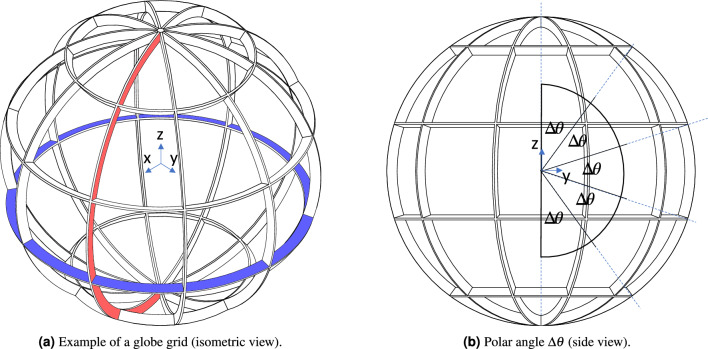
Globe grid with $n_\text {long} = 5$ and $n_\text {lat}=4$ ($N=50$) as an example to illustrate the geometric properties. In (**a**) a single longitude element is highlighted in red and a latitude element is shown in blue. In (**b**) the equiangular distribution of the latitudinal segments based on the polar angle $\Delta \theta$ is highlighted.

Globe grids comprise latitudinal and longitudinal bridge segments as shown in Fig. 11a. The following equation returns the number of apertures $N_\text {globe}$ of a globe grid with $n_\text {lat}$ latitude rings and $n_\text {long}$ longitude rings assuming that the apertures closest to the “north” and “south pole” have a triangular shape and the grid is connected to the conductor rod at the north pole:

12 $$\begin{aligned} N_\text {globe} = 2 n_\text {long} (n_\text {lat}+1), \quad n_\text {long} \ge 1, \ n_\text {lat} \ge 0 \end{aligned}$$

There are two options on how to define the position of the latitude rings:

- The latitude rings are distributed in equidistant steps from the equatorial plane. This has the advantage that the apertures will all have a similar size (this will be shown in the paragraph below). However, the apertures are not equally distributed with regard to the latitude. The arc lengths of the longitude segments of the individual apertures will become larger closer to the “poles”.
- The latitudes are defined by constant polar angles $\Delta \theta$ (see Fig. 11b). This has the advantage that the apertures are more evenly distributed. However, the apertures closer to the poles are smaller than the ones closer to the equator.

The present discussion will only focus on the latter case in which the latitudes are defined by constant polar angles $\Delta \theta$ (see Fig. 11b, since this describes the case in which the apertures are more regularly distributed. To keep the main part concise, the detailed description of the geometry and the calculation of the metrics is given in Appendix A.

### Results of globe grids

**Figure 12:**
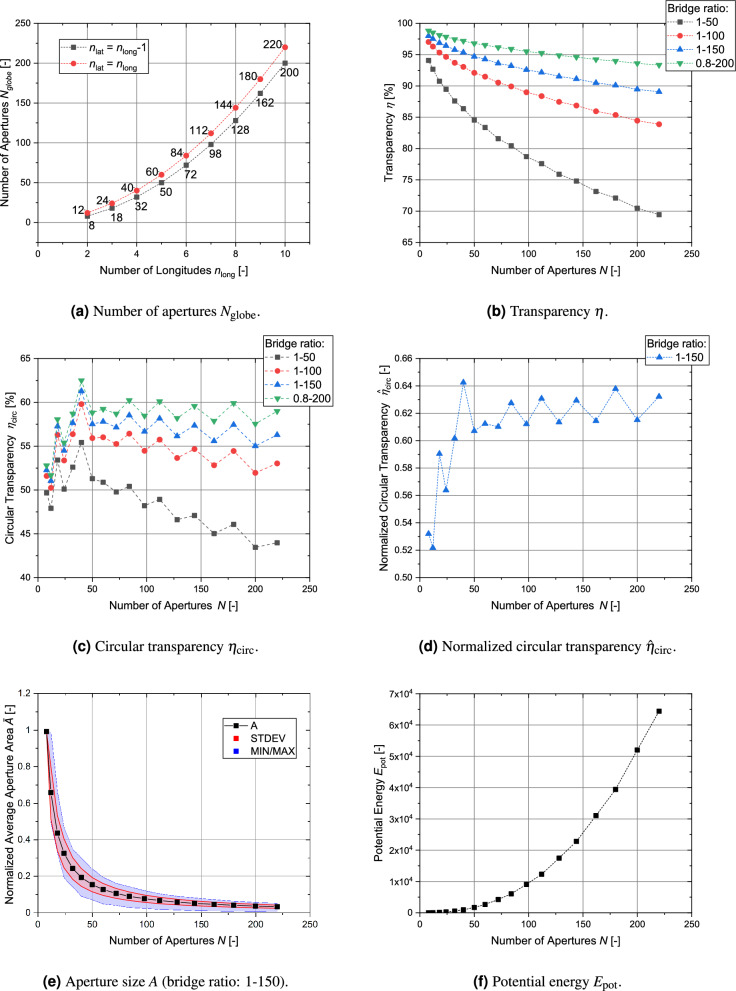
Parameter calculation for globe grids in the range of $N=8$ to 220.

The globe grid does not have a lot of geometrical restrictions and therefore many geometries are possible, e.g. it would be possible to create an (impractical) grid with one single latitudinal segment and 10 longitudinal segments. However, this would lead to very uneven-sized apertures in contradiction with requirement M3. To restrict the analysis to more homogeneously shaped globe grids, only globe grids with $n_\text {lat} \equiv n_\text {long}$ and $n_\text {lat} \equiv n_\text {long}-1$ in the range of $n_\text {long}$ 2 to 10 will be considered. This provides grids with 8 to 220 apertures as displayed in Fig. 12a.

As shown in Fig. 12b globe grids can easily achieve transparencies of more than 90 % for moderate bridge sizes (see Table 2) even at higher aperture numbers. The circular transparency (Fig. 12c ) is significantly lower with values between 50 % to 60 %. The ratio of $n_\text {lat}$ to $n_\text {long}$ plays a significant role, which is illustrated by the “zick zack” behavior of the graphs. Globe grid configurations with the constraint $n_\text {lat} = n_\text {long}$ show higher transparencies. Both the circular transparency and its normalized form (see Fig. 12d) behave very constantly for aperture numbers higher than $N=60$. As shown in Fig. 12e, the area of the aperture initially drops significantly for a higher number of apertures but then the decline becomes smaller. The apertures of the $N=220$ configuration only have about 3 % of the aperture area of the $N=8$ configuration. The potential energy is displayed in Fig. 12f. It gradually increases for higher numbers of apertures. Since this metric is difficult to interpret, it will be analyzed in more detail in Sect. Comparison of parameters of different grid types where all grid types are compared with each other.

## Parameters of symmetric grids

**Figure 13:**
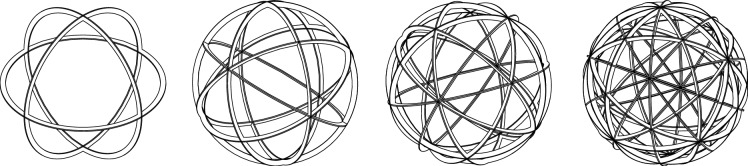
Structure of symmetric grids (made from individual ring-shaped elements). From left to right: 3, 6, 9 and 15 ring elements.

To obtain geometries with the rigorous requirements on same-sized spherical triangle-shaped apertures with antipodal symmetry, only a few geometric configurations based on rings, which in effect represent great circles, are permissible. Ohnishi et al.^37^ presented designs of symmetric grids made from 3, 6 and 9 rings. An overview of symmetric grids with an additional 15-ring grid is given in Table 3 and a graphical representation is shown in Fig. 13.

More complex configurations of this geometry type with additional rings do not seem to be possible. Note how for every configuration two angles are fixed to 90^∘^ and 60^∘^ respectively. The last angle varies with the number of maximum rings crossing in point $n_\text {ring,cross,max}$ by ${360}^{\circ }/(2n_\text {ring,cross,max})$. As shown in Table 3 this number increases linearly with each successive grid configuration. For more than 15 rings the last angle would theoretically reduce to 30^∘^ or less and, therefore violate the rule that the sum of the angles of proper spherical triangles has to be greater than 180^∘^ and less than 540^∘^. However, no definite proof can be presented here and it might be possible that additional configurations for symmetric grids with a higher number of apertures exist.

The symmetric grids rely on spherical projections of special spherical polyhedra, namely the spherical octahedron (3 rings), the spherical tetrakis hexahedron (6 rings), the spherical disdyakis dodecahedron (9 rings) and the spherical disdyakis triacontahedron (15 ring). Their respective duals describe the center-points of these polyhedra for the calculation of the potential energy of the distribution. Although the symmetric grid type is often found in IECF devices, it seems that the underlying geometry has not been analyzed in detail since to the best knowledge of the authors there is no mention of these polyhedra with their complex geometries.

With the limited number of just four configurations, it was found to be a quick method to determine the aperture surface areas graphically with the help of the CAD software SolidWorks. The surface areas were obtained with the measurement tool for the different configurations for various bridge angles $\alpha _\text {bridge}$. From this, the transparency was calculated. The potential energy was calculated from the permutations of the vertex coordinates given in Table 3.

**Table 3:** Geometric configurations of symmetric grid.

	3 rings	6 rings	9 rings	15 rings
# Apertures *N*	8	24	48	120
Angles of spherical apertures	3 x 90^∘^	90^∘^, 2x 60^∘^	90^∘^, 60^∘^, 45^∘^	90^∘^, 60^∘^, 36^∘^
Max. # of rings in one crossing point $n_\text {ring,cross,max}$	2	3	4	5
Spherical polyhedron	Spherical octahedron	Spherical tetrakis hexahedron	Spherical disdyakis dodecahedron	Spherical disdyakis triacontahedron
Dual spherical polyhedron	Cube	Truncated octahedron	Truncated cuboctahedron	Truncated icosidodecahedron
Vertex coordinates of dual (not normalized)	All permutations: $(\pm 1, \pm 1, \pm 1)$	All permutations: $(0, \pm 1, \pm 2)$	All permutations: $(\pm 1, \pm (1+\sqrt{2})$, $\pm (1+\sqrt{2}))$	Even permutations: $(\pm 1/\Phi ,\pm 1/\Phi , \pm (3+\Phi )$, $(\pm 2/\Phi ,\pm \Phi , \pm (1+2\Phi )$, $(\pm 1/\Phi ,\pm \Phi ^2, \pm (-1+3\Phi )$, $(\pm (2\Phi -1),\pm 2, \pm (2+\Phi ))$, $(\pm \Phi ,\pm 3, \pm 2\Phi )$, $\Phi = (1+\sqrt{5})/2$

**Figure 14:**
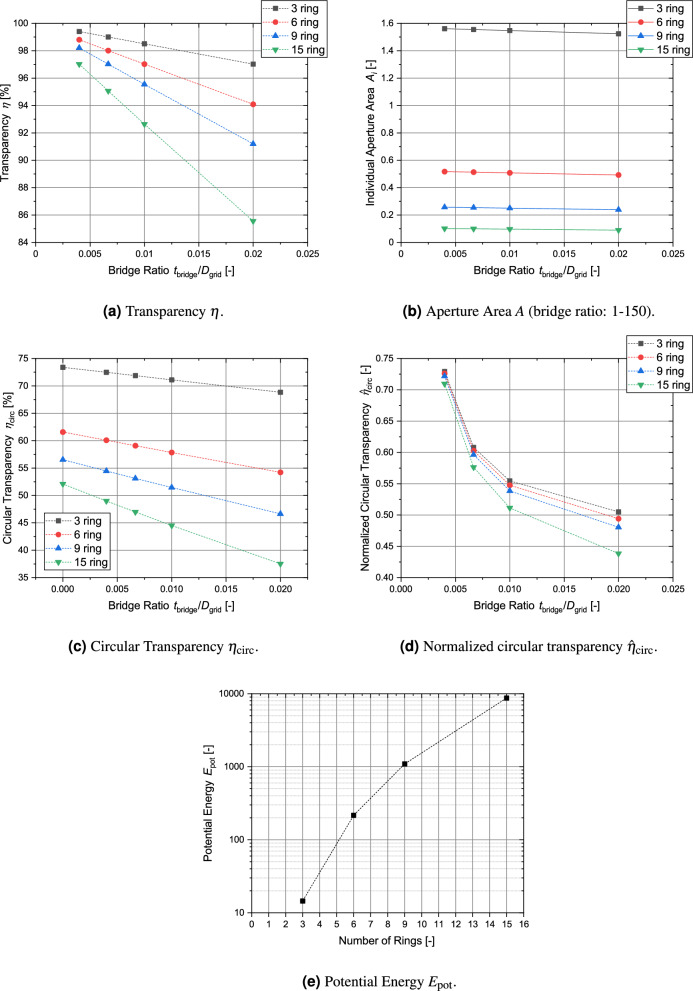
Main parameters for all symmetric grid configurations.

The results of this analysis are visually summarized in Fig. 14. The transparency of this grid type stays well above 90 % with the only exception of the largest grid angle in the 15 ring configuration. The variation of the relative aperture size between the configurations is significant, which is to be expected with the highly different numbers of apertures. The circular transparency also differs between the configurations and becomes smaller for a higher number of rings. It is also significantly influenced by the bridge ratio, as shown in Fig. 14c and 14d. Finally, the potential energy of the grid configurations is given in Fig. 14e.

## Modelling of regular-shaped grid geometries

### Procedure

**Figure 15:**
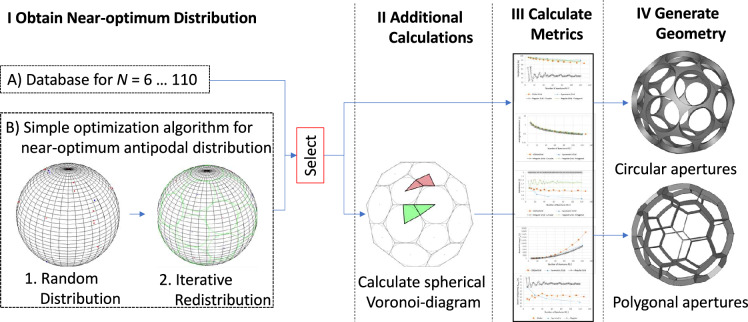
Overview of procedure to generate electrode models and calculate metrics.

The main steps of the procedure for the modelling of the regular-shaped grids are outlined in Fig. 15. The calculation of the geometry is more complex compared to the globe grids and symmetric grids since only for $N = 6$ and 12 a known optimum solution exists. The near-optimum distribution is either obtained from a database^14,15^ or by a simple optimization algorithm, which is presented in Appendix B.2. Based on the distribution the geometry of a regular grid can be generated by defining either circular or polygonal shaped apertures:

- Circular-shaped apertures: The Boolean difference between a unit sphere and a set of spherical caps (see Sect. B.3.1).
- Polygonal-shaped apertures: Boolean difference of the unit sphere and multiple (irregular) convex pyramids with their apexes at the center of the sphere and the centroids on the sphere surface. The polygon of the base of the pyramid is the projection of a Voronoi cell. (see Sect. B.3.2)

**Figure 16:**
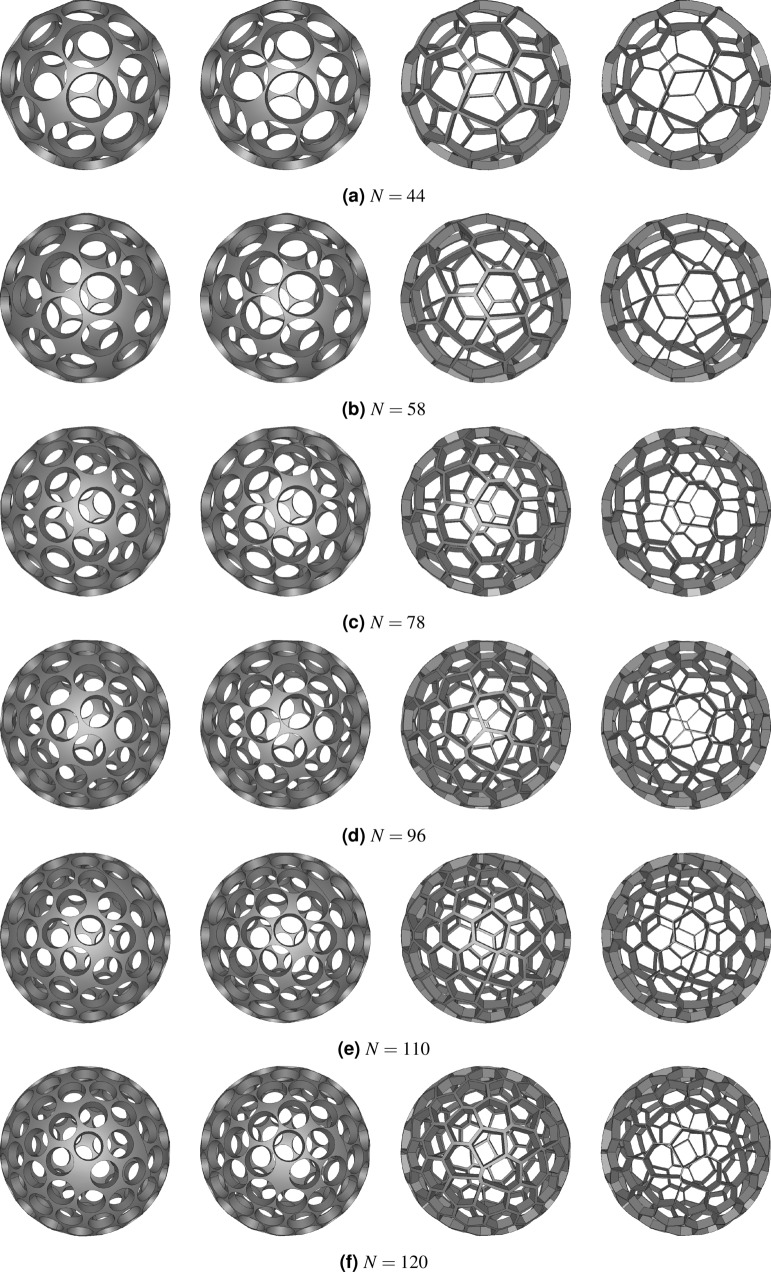
Geometries generated with Salome Meca‘s geometry module. For every configuration the first two grids are of the circular-shaped aperture type and the remaining two of polygonal-shaped aperture type. The bridge-ratio is 1–50 and 1–150.

As in the case of the globe grids, the details for the geometry description and the calculation of the metrics are described in Appendix B. Different configurations of regular-shaped grids with circular and polygonal shaped apertures are shown in Fig. 16. The geometries were procedurally generated with Salome Meca and can be easily exported in file formats suitable for additive manufacturing (e.g. .stl).

### Analysis of results

**Figure 17:**
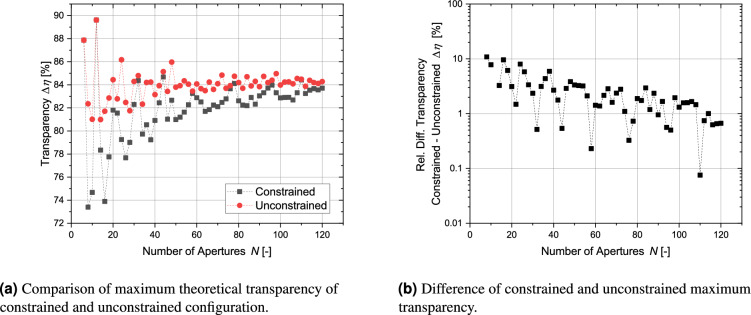
Comparison of transparencies for constrained and unconstrained configurations in the limit of $t_\text {bridge} \rightarrow 0$.

At first, the geometric transparency of the regular-shaped grids with circular apertures was calculated. To see, how the antipodal constraint influenced the transparency, it was compared with the transparency without antipodal constraint. This is shown in Fig. 17a. It can be seen that the antipodal symmetry constraint generally penalizes the transparency compared with the unconstrained case. For the lower range of *N* the transparency generally fluctuates strongly. For higher *N* the fluctuations become smaller. The fluctuations are more pronounced for the antipodal distributions. In the unconstrained case, the following configurations show a high transparency $N = 6, 12, 24, 32, 48, 78, 98, 120$. A high transparency - high in comparison with configurations with a slightly smaller or larger number of apertures - for the unconstrained case does not necessarily correlate with a high transparency for the antipodal constrained case. For example, the transparency of the antipodal configuration $N = 24$ is nearly 7 % smaller. Also, instead of $N = 48$ the value of $N = 44$ delivers better results. A list of promising configurations contains the following values of *N*: 6, 12, 32, 44, 58, 78, 96, 110, 120. A simple way to identify these numbers is to compute the difference between unconstrained and antipodal constrained geometries as displayed in Fig. 17b. It also becomes apparent that for $N = 32$, there is a difference of about 0.52 %. Therefore, the truncated icosahedron does not represent the optimal distribution. In general, some distributions are superficially related to structures of higher symmetry but usually, this requirement has to be relaxed in order to obtain better packaging^38^.

**Figure 18:**
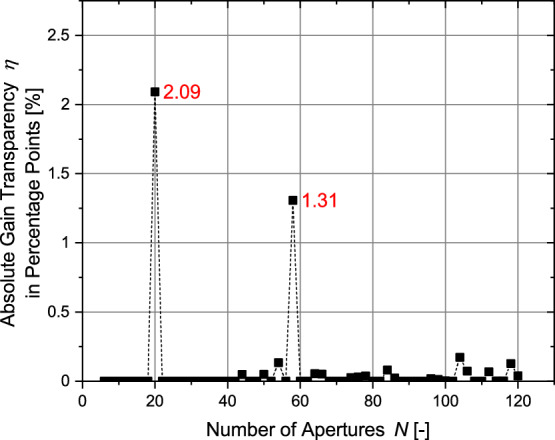
Absolute gain of transparency by maximizing the diameter of individual apertures. Only certain configurations show a significant gain.

The constraint of similar-sized apertures influences the transparency. It becomes apparent from Fig. 18 that only two configurations ($N = 20$ and 58) profit if the diameter of all apertures is maximized by a low gain of more than 1 percentage point.

**Figure 19:**
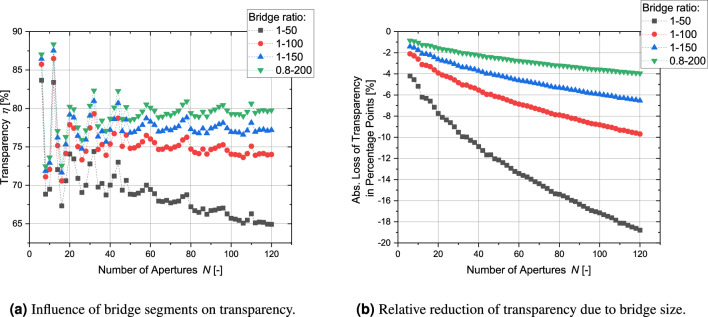
Influence of finite bridge element thickness on regular grids with circular apertures.

In the next step, the influence of the finite-sized bridge elements between the apertures on the overall transparency is analyzed. These bridges necessarily exist for real cathode grids. As shown by Fig. 19 the reduction of transparency is quite significant. However, by looking at Fig. 19b it becomes apparent that the relative reduction of transparency decreases for a higher number of apertures. A grid with 120 apertures and a bridge-ratio of 1 to 150 can still have a transparency of roughly 77 %.

**Figure 20:**
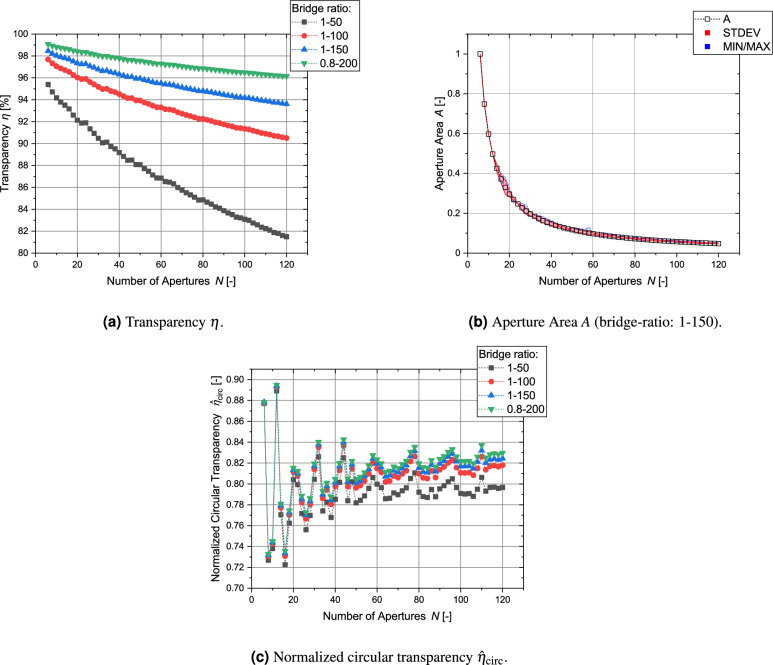
Main parameters for all configurations of regular-shaped grids with polygonal apertures (for circular transparency $\eta _\text {circ}$ see identical values of transparency $\eta$ of regular-shaped grids with circular apertures (Fig. 19)).

So far, the transparency of electrodes with circular apertures has been discussed. The next paragraph focuses on the regular-shaped grids with polygonal apertures: Fig. 20a shows the transparency for grids with polygonal apertures. Because the polygonal structure maximizes the cut-outs in the spherical structure, the transparency is significantly higher. Even for a bridge-ratio of 1-50, the transparency for 120 apertures is still above 80 % and therefore higher than for nearly all configurations with circular apertures and a bridge-ratio of just 1–150. Thus, this type of geometry allows experiments in IECF fusion devices with transparencies close to that of globe grids and symmetric grids. The variety between the individual aperture sizes is small (see Fig. 20b). It should be kept in mind that these values were calculated for 1 out of 10 iterations of which the iteration with the highest transparency was chosen. It is possible that values for other parameters might differ significantly depending on the numerically optimized distribution. According to Fig. 20c, the values for the normalized circular transparency are well within the range of 0.72 to 0.9 and close to 0.8 for higher *N* with a small influence of the brdige-ratio. Table 4 compares the transparencies of regular-shaped grids with circular apertures and with polygonal apertures.

**Table 4:** Comparison of the most relevant configurations of regular-shaped grids for different bridge ratios $t_\text {bridge}/D_\text {grid}$.

	Transparency [%]								
	Circular apertures					Polygonal apertures			
*N*	–	1–50	1–100	1–150	0.8–200	1–50	1–100	1–150	0.8–200
6	87.87	83.67	85.76	86.46	87.02	95.39	97.68	98.45	99.07
12	89.61	83.40	86.48	87.52	88.35	93.79	96.87	97.91	98.74
32	84.36	74.41	79.31	80.98	82.32	90.07	94.97	96.63	97.97
44	84.68	73.01	78.74	80.69	82.28	88.48	94.15	96.08	97.64
58	83.25	70.02	76.50	78.72	80.52	86.86	93.31	95.51	97.30
78	84.12	68.77	76.25	78.83	80.93	84.84	92.26	94.81	96.87
96	83.97	67.04	75.27	78.12	80.44	83.28	91.45	94.25	96.53
110	84.42	66.30	75.09	78.14	80.62	82.24	90.90	93.88	96.31
120	83.71	64.93	74.02	77.19	79.77	81.50	90.51	93.62	96.15

The maximum value is taken from 10 iterations for the iterative optimization of the underlying distribution.

## Comparison of parameters of different grid types

**Figure 21:**
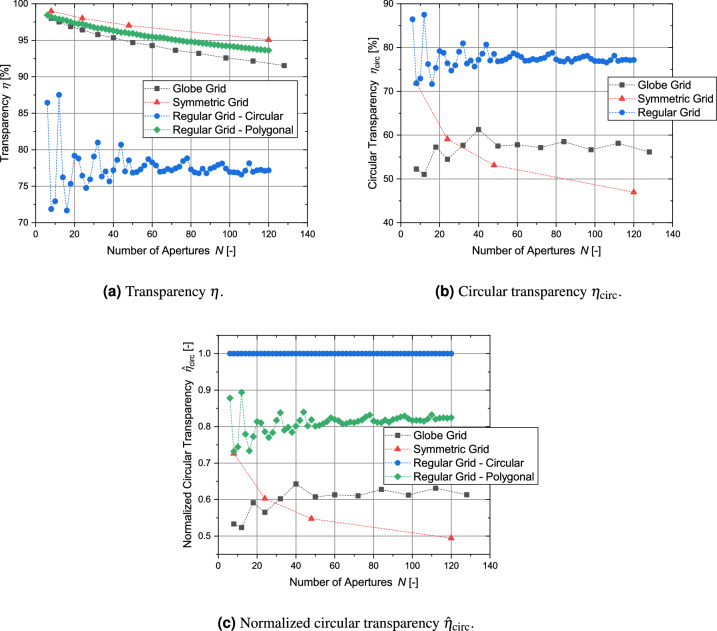
Comparison of the geometric transparency $\eta$, the circular transparency $\eta _\text {circ}$ and the normalized circular transparency ${\hat{\eta }}_\text {circ}$ for the different types of grid geometries (bridge ratio $t_\text {bridge}/D_\text {grid}=1-150$).

**Figure 22:**
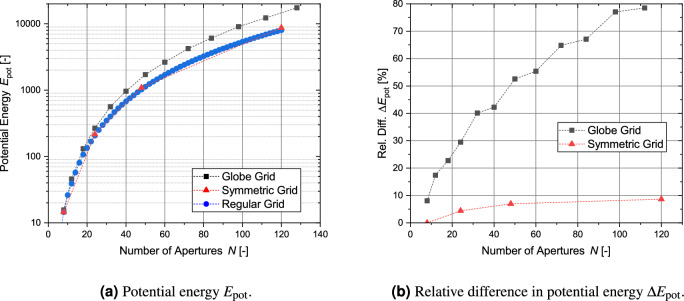
Comparison of the potential energy $E_\text {pot}$ for the different types of grid geometries.

**Figure 23:**
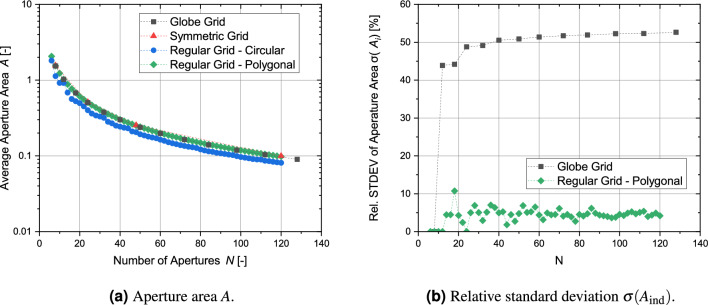
Comparison of the individual aperture areas $A_\text {ind}$ for the different types of grid geometries.

This section compares the results of the analysis of the four different metrics for the globe grids, symmetric grids and regular-shaped grids. For a clear presentation of the results, only the bridge ratio of 1/150 is analyzed. All grid configurations with the exception of the regular grid with circular apertures achieve transparencies over 90 % (see Fig. 21a) and the differences between the grid types are small with the symmetric grid showing the best values. However, in the case of the circular transparency $\eta _\text {circ}$ and its normalized form, the order is essentially reversed (see Fig. 21b and c). Figure 22 shows the results for all grid configurations with the regular shapes having the overall lowest potential energy, as their geometry is specifically optimized for this. For $N=120$, the symmetric grid has a 8.6 % higher potential energy, while the globe grid configuration for $N=112$ has a nearly 80 % higher potential energy. The homogeneity of the aperture areas is compared in Fig. 23. The average size is similar for all configurations. Only the circular apertures lead to a significantly reduced aperture area. In the case of the symmetric grids and the regular grids with circular apertures, all apertures have the same size. The variation between the individual aperture sizes for the two remaining grid types is displayed in Fig. 23b. The aperture areas vary significantly more for globe grids than for regular grids with polygonal apertures.

Table 5 provides a qualitative comparison of the different grid types in relation to the four metrics.

**Table 5:** Qualitative comparison of metrics for different grid types (+ good, O neutral, - bad).

#	Metric	Globe grids	Symmetric grids	Regular grids (circular apertures)	Regular grids (polygonal apertures)
M1	Transparency $\eta$	+	+	O	+
M2	Homogeneity of aperture $\sigma (A_i)$	–	+	+	O
M3	Circular transparency $\eta _\text {circ}$	+	+	O	+
M4	Distribution of apertures over grid structure $E_p$	–	O	+	+

## Summary and outlook

A detailed analysis of the geometries of cathode grids that are found in inertial electrostatic confinement fusion devices was conducted. Based on a brief literature review, three different types of geometries were identified: Globe grids, symmetric grids and regular-shaped grids. While the former are common and have been widely tested, the latter constitute a novel and scarcely explored class of grid geometries. In addition, the regular grids can be distinguished into regular grids with circular apertures and polygonal apertures. For each type of grid, a definition of the geometry was presented. All types of grids were compared based on a set of four metrics in the range of 6 to 120 apertures. It was found that all types of grids come with different advantages and disadvantages. For example, all types of grids with the exception of the regular shaped grids with circular apertures can achieve transparencies over 90 %. But regular grids can have the highest circular transparency whereas globe grids and symmetric grids fall short. Regular grids and symmetric grids feature similar-sized apertures and regular grids have the best distribution of the apertures. In the case of the regular shaped grids with circular apertures, in which the transparency is generally the lowest, a set of configurations was identified which might be of interest for future tests in IECF devices ($N = 6, 12, 32, 44, 58, 78, 96, 110$ and 120.). At the moment, the presented geometric metrics with the exception of the transparency cannot be directly related to the performance of IECF devices due to the lack of experimental data. Therefore, a dedicated test campaign covering multiple versions of all grid types is required to establish a link between geometric metrics and performance metrics.

### Supplementary Information

Supplementary Information.

## Data Availability

The datasets used and analysed during the current study are available from the corresponding author on reasonable request.

## References

[1] Bussard RW (1991). Some physics considerations of magnetic inertial-electrostatic confinement: A new concept for spherical converging-flow fusion. Fusion Technol..

[2] Barnes DC, Nebel RA (1998). Stable, thermal equilibrium, large-amplitude, spherical plasma oscillations in electrostatic confinement devices. Phys. Plasmas.

[3] Nebel RA, Barnes DC (1998). The periodically oscillating plasma sphere. Fusion Technol..

[4] Dietrich, C.C. *Improving particle confinement in inertial electrostatic fusion for spacecraft power and propulsion by*. Dissertation, Massachusetts Insitute of Technology (2007).

[5] Chap, A. M. *Simulation and optimization of the continuous electrode inertial electrostatic confinement fusor*. Dissertation, University of Maryland (2017).

[6] Fancher, A. N. *Fusion Neutron production using deuterium fuel in an inertial electrostatic confinement device at 10 to 200 Kilovolts*. Dissertation, University of Wisconsin-Madison (2018).

[7] Radel, R. F. *Detection of Highly Enriched Uranium and Tungsten Surface Damage Studies Using a Pulsed Inertial Electrostatic Confinement Fusion Device*. Ph.D. thesis, University of Wisconin-Madison (2007). 10.13182/FST52-1087.

[8] Miley GH, Murali SK (2014). Inertial Electrostatic Confinement (IEC) Fusion.

[9] Wulfkuehler, J.-P. & Tajmar, M. Novel Inertial Electrostatic Confinement Fusion with Buckyball-Shaped Multi-Grids. In *52nd AIAA/SAE/ASEE Joint Propulsion Conference* (American Institute of Aeronautics and Astronautics, 2016), 10.2514/6.2016-4777.

[10] Bowden-Reid R, Khachan J, Wulfkühler JP, Tajmar M (2018). Evidence for surface fusion in inertial electrostatic confinement devices. Phys. Plasmas.

[11] Bakr M (2021). Evaluation of 3D printed buckyball-shaped cathodes of titanium and stainless-steel for IEC fusion system. Phys. Plasmas.

[12] Rasmussen J (2020). Characterization of fusion plasmas in the cylindrical DTU inertial electrostatic confinement device. Phys. Plasmas.

[13] Bakr, M. *et al.* Influence of electrodes geometrical properties on the neutron production rate of a discharge fusion neutron source #526. In poster session SOFE 2021.

[14] Sloane, N. J. A. Spherical Codes. Nice arrangements of points on a sphere in various dimensions. Accessed 25 January 2023. http://neilsloane.com/packings/index.html.

[15] Conway JH, Hardin RH, Sloane NJA (1996). Packing lines, planes, etc. Packings in Grassmannian spaces. Exp. Math..

[16] Gautam, S. & Vaintrob, D. A Novel Approach to the Spherical Codes Problem.

[17] Wehmeyer AL, Radel RF, Kulcinski GL (2005). Optimizing neutron production rates from D-D fusion in an inertial electrostatic confinement device. Fusion Sci. Technol..

[18] Dolan TJ, Verdeyen JT, Meeker DJ, Cherrington BE (1972). Electrostatic-inertial plasma confinement. J. Appl. Phys..

[19] Black WM, Robinson JW (1974). Measuring rotationally symmetric potential profiles with an electron-beam probe. J. Appl. Phys..

[20] Miley G (1997). Discharge characteristics of the spherical inertial electrostatic confinement (IEC) device. IEEE Trans. Plasma Sci..

[21] Miley GH, Sved J (1997). The IEC-A plasma-target-based neutron source. Appl. Radiat. Isot..

[22] Miley GH (2013). Life at the Center of the Energy Crisis: A Technologist’s Search for a Black Swan.

[23] Murali SK, Kulcinski GL, Santarius JF (2008). Study of ion flow dynamics in an inertial electrostatic confinement device through sequential grid construction. Phys. Plasmas.

[24] Michalak MK, Fancher AN, Kulcinski GL, Santarius JF (2017). Expanding operational space in inertial electrostatic confinement D-D neutron generators. Fusion Sci. Technol..

[25] Bakr M, Mukai K, Masuda K, Yagi J, Konishi S (2021). Characterization of an ultra-compact neutron source based on an IEC fusion device and its prospective applications in radiography. Fusion Eng. Des..

[26] Hirsch RL (1968). Experimental studies of a deep, negative, electrostatic potential well in spherical geometry. Phys. Fluids.

[27] Ulmen, B. *Formation and Extraction of a Dense Plasma Jet from a Helicon-Injected-Inertial Electrostatic Confinement Device*. Ph.D. thesis, University of Illinois at Urbana-Champaign (2013).

[28] Bowden-Reid R, Khachan J (2021). An inertial electrostatic confinement fusion system based on graphite. Phys. Plasmas.

[29] Bakr M, Masuda K, Yoshida M (2019). Development of a portable neutron generator based on inertial electrostatic confinement D-D fusion reaction. AIP Conf. Proc..

[30] McGuire, T. *Improved Lifetimes and Synchronization Behavior in Multi-grid Inertial Electrostatic Confinement Fusion Devices*. Dissertation, Massachussetts Institut (2007).

[31] Sedwick, R. Magnetic Core Multi-Grid IEC Fusion as an Entry Point to a Hydrogen-Based Economy. In *8th Annual International Energy Conversion Engineering Conference*, 10.2514/6.2010-6605 (American Institute of Aeronautics and Astronautics, Reston, 2010).

[32] Murali SK, Cipiti BB, Santarius JF, Kulcinski GL (2006). Study of fusion regimes in an inertial electrostatic confinement device using the new eclipse disk diagnostic. Phys. Plasmas.

[33] Chan, Y.-A. & Herdrich, G. Back-vacuum Retarding Potential Analyzer for Investigation Plasma Properties from Inertial Electrostatic Confinement Thruster. In *36th International Electric Propulsion Conference*, IEPC-2019-292 (2019).

[34] Bakr M, Masuda K, Yoshida M (2019). Improvement of the neutron production rate of IEC fusion device by the fusion reaction on the inner surface of the IEC chamber. Fusion Sci. Technol..

[35] Thorson TA, Durst RD, Fonck RJ, Wainwright LP (1997). Convergence, electrostatic potential, and density measurements in a spherically convergent ion focus. Phys. Plasmas.

[36] Saff EB, Kuijlaars ABJ (1997). Distributing many points on a sphere. Math. Intell..

[37] Ohnishi M, Osawa H, Tanaka R, Wakizaka N (2005). Shape of electrodes for high performance of inertial electrostatic confinement fusion. J. Nuclear Sci. Technol..

[38] Clare BW, Kepert DL (1991). The optimal packing of circles on a sphere. J. Math. Chem..

