# Supplementary material for: A systematic approach to the modelling and comparison of the geometries of spherical electrodes in inertial electrostatic confinement fusion devices
Source: Sci Rep. 2024 Jan 27;14:2261. doi: 10.1038/s41598-024-52173-6 (PMC10817989; doi:10.1038/s41598-024-52173-6)
Supplement: Supplementary file 1 — Supplementary Information. [file 41598_2024_52173_MOESM1_ESM.pdf]

Appendices

A Globe Grids - Detailed Parameter Calculation

A.1 Calculation of Geometry

For the calculation of the grid coordinates it is beneficial to use spherical coordinates wherever possible. Each aperture can be associated with a constant polar angle $\Delta\theta$ and a constant azimuthal angle $\Delta\varphi$, which can be calculated from the numbers of longitudinal segments n_{long} and latitudinal segments n_{lat}:

$$\Delta\theta = \frac{\pi}{n_{\text{lat}}+1} \tag{A.1}$$

$$\Delta\varphi = \frac{\pi}{n_{\text{long}}} \tag{A.2}$$

As it can be seen from Fig. A.1, the geometry of the apertures close to the poles diverges from the other ones. Real cathode grids in IEC devices might feature a slightly different alignment of the longitudinal segments at the top and bottom. In order to not lose the generality of the approach presented here, it is assumed that all longitudinal elements cross at the top and bottom. The variable ξ will be used to enumerate the apertures from top to bottom with $\xi = 1\ldots(n_{\text{lat}}+1)$ (see Fig. A.1a and analogously as shown in Fig. A.1b $\nu = 1\ldots 2n_{\text{long}}$ in the azimuthal direction). The top region (as displayed in Fig. A.1c) can be described by the top radius r_{top} and polar top angle θ_{top}, which are defined by:

$$r_{\text{top}} = R_{\text{grid}} \frac{\sin\alpha_{\text{bridge}}}{\sin\left(\frac{\Delta\varphi}{2}\right)} \tag{A.3}$$

$$\theta_{\text{top}} = \arcsin\left(\frac{r_{\text{top}}}{R_{\text{grid}}}\right) \tag{A.4}$$

For the description of the aperture coordinates, two sets of points $\hat{\mathbf{v}}_{\text{globe,up}}$ and $\hat{\mathbf{v}}_{\text{globe,low}}$ will be used (see Fig. A.1d). They describe one-half of the corner points of the apertures which are assumed to be symmetric to the x-z plane. The apertures closest to the poles are triangles. The corner points are defined by the intersection of the longitudinal elements (see also the detailed top-view in Fig. A.1c with $\hat{\mathbf{v}}_{\text{globe,up}}(\xi, \nu = 1)$). Therefore, the upmost corner point has the following coordinates.

$$\hat{\mathbf{v}}_{\text{globe,up}}(\xi = 1) = \begin{cases} x_{\text{up}} = R_{\text{grid}}\cos(\theta_{\text{top}}) \\ y_{\text{up}} = 0 \\ z_{\text{up}} = R_{\text{grid}}\sin(\theta_{\text{top}}) \end{cases} \tag{A.5}$$

For the calculation of the trapezoidal coordinates the three auxiliary variables $x'_{\text{up}}, y'_{\text{up}}$ and z'_{up} are defined first:

$$z'_{\text{up}} = R_{\text{grid}}\cos((\xi-1)\Delta\theta + \alpha_{\text{bridge}}) \tag{A.6}$$

$$x'_{\text{up}} = \sqrt{(R_{\text{grid}}\cos(\alpha_{\text{bridge}}))^2 - (z'_{\text{up}})^2} \tag{A.7}$$

$$y' = -R_{\text{grid}}\sin(\alpha_{\text{bridge}}) \tag{A.8}$$

These can now be used to calculate the remaining upper aperture coordinates:

$$\hat{\mathbf{v}}_{\text{globe,up}}(\xi) = \begin{cases} x_{\text{up}} = x'_{\text{up}}\cos\left(\frac{\Delta\varphi}{2}\right) - y'\sin\left(\frac{\Delta\varphi}{2}\right) \\ y_{\text{up}} = y'\cos\left(\frac{\Delta\varphi}{2}\right) + x'_{\text{up}}\sin\left(\frac{\Delta\varphi}{2}\right) \\ z_{\text{up}} = z'_{\text{up}} \end{cases} \quad, \xi = 2 \ldots n_{\text{lat}}+1 \tag{A.9}$$

Analogously, the lower coordinates can be calculated. Only two of the auxiliary variables need to be adapted:

$$z'_{\text{low}} = R_{\text{grid}} \cos\left((\xi - 1)\Delta\theta - \alpha_{\text{bridge}}\right) \tag{A.10}$$

$$x'_{\text{low}} = \sqrt{(R_{\text{grid}} \cos(\alpha_{\text{bridge}}))^2 - (z'_{\text{low}})^2} \tag{A.11}$$

This leads to:

$$\hat{\mathbf{v}}_{\text{globe,low}}(\xi) = \begin{cases} x_{\text{low}} = x'_{\text{low}} \cos\left(\frac{\Delta\varphi}{2}\right) - y' \sin\left(\frac{\Delta\varphi}{2}\right) \\ y_{\text{low}} = y' \cos\left(\frac{\Delta\varphi}{2}\right) + x'_{\text{low}} \sin\left(\frac{\Delta\varphi}{2}\right) \\ z_{\text{low}} = z'_{\text{low}} \end{cases}, \ \xi = 1 \ldots n_{\text{lat}} \tag{A.12}$$

For the aperture closest to the south pole the following coordinates apply:

$$\hat{\mathbf{v}}_{\text{globe,low}}(\xi = n_{\text{lat}} + 1) = \begin{cases} x_{\text{low}} = R_{\text{grid}} \cos(\pi - \theta_{\text{top}}) \\ y_{\text{low}} = 0 \\ z_{\text{low}} = R_{\text{grid}} \sin(\theta_{\text{top}}) \end{cases} \tag{A.13}$$

A.2 Calculation of Transparency

The calculation of the transparency is as follows: From the surface area of a spherical zone, it can be derived that an individual aperture, which is in an azimuthal direction essentially described by the azimuthal angle $\Delta\theta$ (additionally reduced by the bridge angle α_{bridge}) and in polar direction by the z-coordinates of $\hat{\mathbf{p}}_{\text{globe,up}}$ and $\hat{\mathbf{p}}_{\text{globe,low}}$ (which both directly depend on the polar angle $\Delta\theta$ and the influence of the bridge angle α_{bridge}). The individual surface area of one aperture can therefore be described by:

$$A_{\text{ap,globe,ind}}(\xi) = 2R_{\text{grid}} \int_{z_{\text{low}}(\xi)}^{z_{\text{up}}(\xi)} \left(\frac{\Delta\varphi}{2} - \arcsin\left(\frac{\frac{t_{\text{bridge}}}{2}}{\sqrt{R^2 - z^2}} \right) \right) dz \tag{A.14}$$

This equation can be simply solved numerically (e.g. with Matlab 2021b‘s *integral*-function). To obtain the transparency of the globe grid, the surface area of all apertures needs to be computed and divided by the surface area of the sphere that has the same diameter as the grid:

$$\eta_{\text{globe-grid}} = \frac{2n_{\text{long}} \sum_{\xi=1}^{n_{\text{lat}}+1} A_{\text{ap,globe,ind}}(\xi)}{4\pi R_{\text{grid}}^2} \tag{A.15}$$

A.3 Calculation of Potential Energy

The potential energy of the globe grid is computed from the center-points of which each is associated with an individual aperture by the half of the polar angle θ. Although this is different from the centroid defined by four corner points and also different from the centroid of the surface area, these coordinates describe the position of the individual apertures of the globe grid and will therefore be used for the metric. (In the case of the symmetric grids and regular shaped grids with polygonal apertures the centroid defined by the corner points is actually the centroid that defines the center of the aperture).
The position of the center points $\hat{\mathbf{p}}_{\text{globe,ap}}$ of the apertures are described in spherical coordinates by (see Fig. A.1d):

$$\hat{\mathbf{p}}_{\text{globe,ap}}(\xi, \nu) = \begin{cases} r = R_{\text{grid}} \\ \theta = (\xi - 1)\Delta\theta + \frac{1}{2}\Delta\theta, \quad \xi = 1 \ldots n_{\text{lat}} + 1 \\ \varphi = (\nu - 1)\Delta\varphi + \frac{1}{2}\Delta\varphi, \quad \nu = 1 \ldots 2n_{\text{long}} \end{cases} \tag{A.16}$$

These points are then used to calculate the potential energy with Eq. 11.

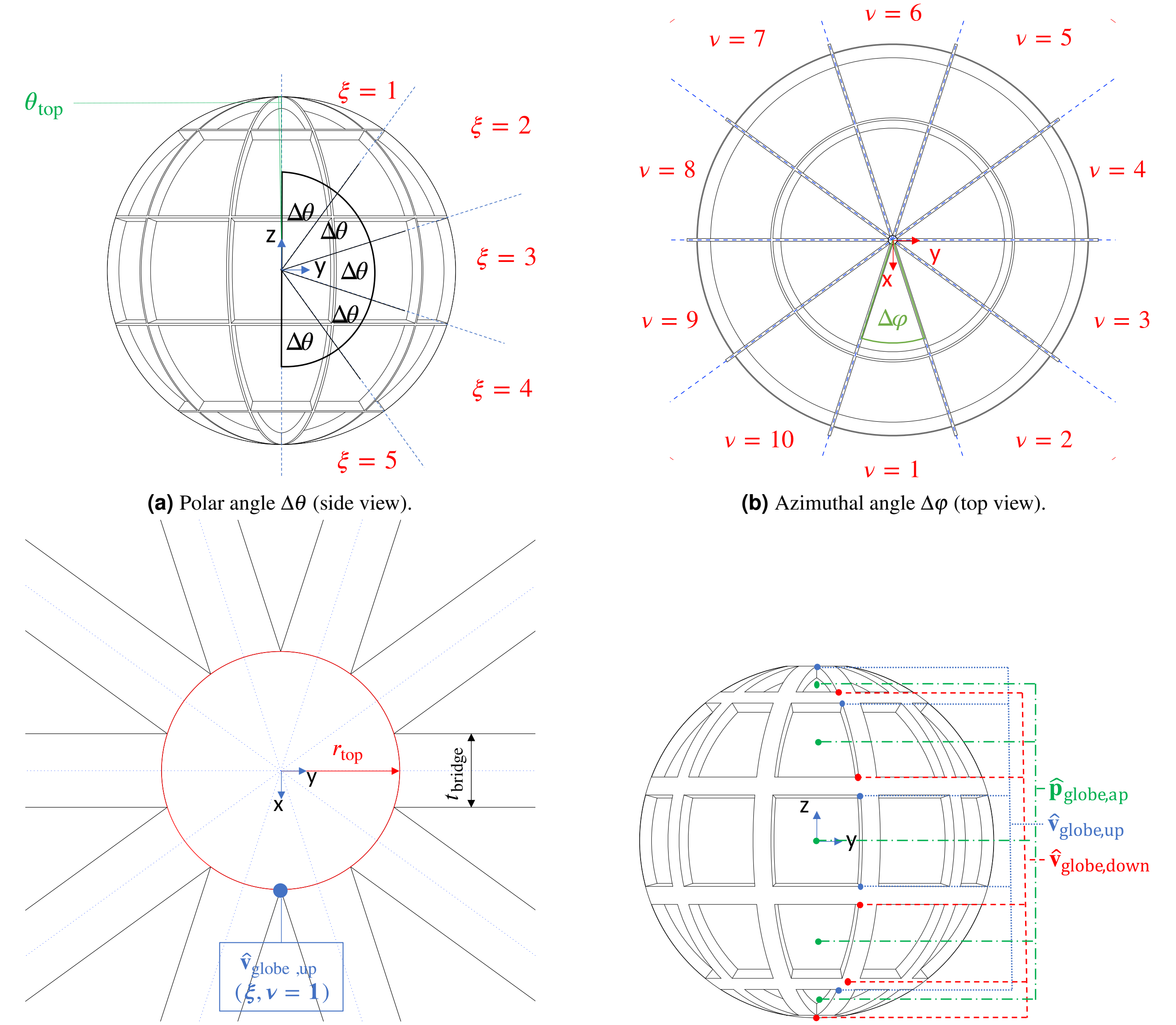

(a) Polar angle $\Delta\theta$ (side view).

(b) Azimuthal angle $\Delta\varphi$ (top view).

(c) Intersection of longitudinal elements at "north" pole (detailed top view).

(d) Aperture Coordinates (side view, t_{bridge} increased for visual clarity).

Figure A.1. Geometric properties of globe grid with conformal cross-section and equiangular distribution of latitudinal segments by constant polar angle $\Delta\theta$ (see **(a)**) and equiangular distribution of longitudinal segments by constant azimuthal angle $\Delta\varphi$ (see **(b)**). **(c)** shows detailed view of the intersection at the top section, which is described by the top radius r_{top} and top angle θ_{top}. **(d)** describes the centerpoints of apertures $\hat{\mathbf{p}}_{globe,ap}$ as well as the vertices of the cornerpoints of the apertures $\hat{\mathbf{v}}_{globe,up}$ and $\hat{\mathbf{v}}_{globe,down}$.

A.4 Calculation of Circular Transparency

The circular transparency of the globe grid $\eta_{circ,globe}$ is calculated following the general approach described in Chapter 4. A visualization is presented in Fig. A.2. For each aperture, the minimum angle between its centroid (see Eq. A.16) and the bridge element is used to compute an angle for a spherical cap:

$$\theta_{cap,circ}(\xi,\nu) = \theta_{min} - \alpha_{bridge} = \frac{\min\left(\Delta\theta, \arccos\left(\frac{\hat{\mathbf{p}}_{globe,ap}(\xi,\nu)\cdot\hat{\mathbf{p}}_{globe,ap}(\xi,\nu+1)}{R_{grid}^2}\right)\right)}{2} - \alpha_{bridge} \tag{A.17}$$

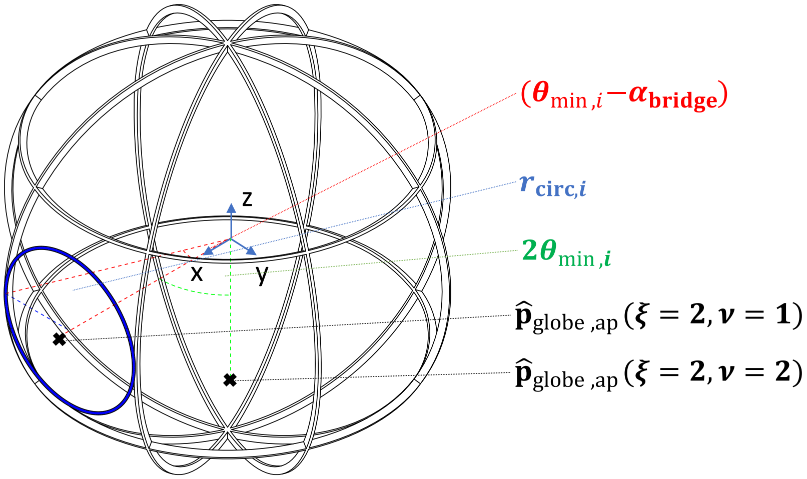

Figure A.2. Illustration of circular transparency η_{circ} for globe grid configurations. The outline of the spherical cap with base radius r_{circ} (with half-angle $(\theta_{\min} - \alpha_{\text{bridge}})$) and its centerpoint $\hat{\mathbf{p}}_{\text{globe,ap}}(\xi = 3, \nu = 1)$ is highlighted by the blue ring.

From the equation for the surface area of a spherical cap follows:

$$A_{\text{cap,circ},i} = 2\pi R_{\text{grid}}^2 (1 - \cos(\theta_{\text{cap,circ}})) \tag{A.18}$$

Therefore, the circular transparency of the globe grid $\eta_{\text{globe,circ}}$ becomes:

$$\eta_{\text{globe,circ}} = \frac{\sum_{i=1}^{N} A_{\text{cap,circ},i}}{4\pi R_{\text{grid}}^2} \tag{A.19}$$

The normalized circular transparency of the globe grids $\hat{\eta}_{\text{globe,circ}}$ is calculated analogously to Eq. 10.

B Regular Grids - Detailed Parameter Calculation

B.1 Mathematical Problems Related to Distribution Problem

Before the iterative optimization procedure to achieve a near-optimum aperture distribution is described, a brief overview of related mathematical problems is presented in the following list:

- Tammes Problem: Motivated from the distribution of pores on pollen grains, this problem asks for the optimum distribution of equally sized circles on a sphere such that their radius becomes maximized without overlapping[1]. In the present case, the circles represent the base of the spherical caps which define the apertures.
- Thomson Problem: To describe the electron distribution in the superseded "plum pudding model" of the atom, Thomson looked for the distribution of N electrons on the surface of a unit sphere in which the overall electrostatic potential energy (based on the Coulomb repulsion between the electrons, see Eq. 11) becomes minimal[2]. In the context of this study, the position of the electrons describes the center of the apertures.
- Spherical Codes: This is somewhat of an inversion to the two problems above and describes a set of N points on an Euclidean sphere with a defined minimum Euclidean distance. Whereas spherical codes are based on a predefined minimum Euclidean distance, the present problem in this paper requires the identification of a configuration such that the Euclidean distance between a set of points is maximized[3].

B.2 Aperture Distribution Optimization

The optimization of the distribution follows a three-step process as described by Gautam and Vaintrob[4]. 1. Initially, the set of points $\hat{\mathbf{p}}_i$ which describes the centroids of the apertures is randomly distributed over the surface of the sphere. 2. Then, the points are iteratively redistributed by the gradient of their potential energy. 3. Finally, the points are iteratively optimized by the individual maximization of the angles between the two closest neighbors. Similar approaches are also described in[5,6].

For the initial random distribution of the points $\hat{\mathbf{p}}_i$ over the surface of the sphere, it was found to be useful to define the position of two apertures, e.g. two being on the z-axis ("north" and "south" pole).

$$\hat{\mathbf{p}}_1 = (0,0,1), \quad \hat{\mathbf{p}}_{N/2+1} = (0,0,-1) \tag{B.20}$$

It can be advantageous to align one or more additional points with a reference such as the x-z plane. In the second step the gradient of the potential energy E_{pot} defined in Eq. 11 is used to redistribute the points:

$$\nabla E_{\text{pot}}(\hat{\mathbf{p}}_i) = \left(\sum_{i \neq j}^{N} \frac{-2s(x_i - x_j)}{||\hat{\mathbf{p}}_i - \hat{\mathbf{p}}_j||^{s+1}}, \sum_{i \neq j}^{N} \frac{-2s(y_i - y_j)}{||\hat{\mathbf{p}}_i - \hat{\mathbf{p}}_j||^{s+1}}, \sum_{i \neq j}^{N} \frac{-2s(z_i - z_j)}{||\hat{\mathbf{p}}_i - \hat{\mathbf{p}}_j||^{s+1}} \right) \tag{B.21}$$

The points $\hat{\mathbf{p}}_i$ are then redistributed by:

$$\hat{\mathbf{p}}_i \rightarrow \frac{\hat{\mathbf{p}}_i - \varepsilon_1 \cdot \nabla E_{\text{pot}}(\hat{\mathbf{p}}_i)}{||\hat{\mathbf{p}}_i - \varepsilon_1 \cdot \nabla E_{\text{pot}}(\hat{\mathbf{p}}_i)||} \tag{B.22}$$

The factor ε_1 weighs the gradient and Gautam[4] suggests $\varepsilon_1 \approx 10^{-3}$.

The next step of the optimization process involves the identification of the closest neighbor $\hat{\mathbf{p}}_j$ for each point $\hat{\mathbf{p}}_i$ and both points are relocated in opposite directions. The closest neighbor for each point is identified by the dot product:

$$d(\hat{\mathbf{p}}_i, \hat{\mathbf{p}}_j) = \arccos(\hat{\mathbf{p}}_i \cdot \hat{\mathbf{p}}_j) \tag{B.23}$$

The subsequent optimization is then achieved by moving each pair of closest points - here denoted as $\hat{\mathbf{p}}_1$ and $\hat{\mathbf{p}}_1$ into the opposite direction with the weighting function ε_2:

$$\hat{\mathbf{p}}_1 \rightarrow \frac{\hat{\mathbf{p}}_1 - \varepsilon_2 \hat{\mathbf{p}}_2}{||\hat{\mathbf{p}}_1 - \varepsilon_2 \hat{\mathbf{p}}_2||} \tag{B.24}$$

$$\hat{\mathbf{p}}_2 \rightarrow \frac{\hat{\mathbf{p}}_2 - \varepsilon_2 \hat{\mathbf{p}}_1}{||\hat{\mathbf{p}}_2 - \varepsilon_2 \hat{\mathbf{p}}_1||} \tag{B.25}$$

If necessary, the antipodal requirement has to be taken into account by realigning the distribution by Eq. 4. Gautam[4] suggests the following function which decreases with the number of optimization steps n_{dot}:

$$\varepsilon_2(n_{\text{dot}}) = \exp\left(\frac{-n_{\text{dot}}^{3/4}}{500} \right) \tag{B.26}$$

B.3 Definition of Apertures and Calculation of Transparency

B.3.1 Circular Apertures

The circular apertures are described by conical cuts into the unit sphere. The surface areas of these apertures are that of spherical caps, which are defined by the half-angle θ. As stated in Section 2.2, it is recommended that all the apertures should have the same size. Therefore, it is necessary to find the minimum angle between the closest apertures by using Eq. B.23. The minimum half angle θ_{min} is one half of this angle (see also Fig. 7).

$$\theta_{\text{min}} = \frac{\min(\arccos(\hat{\mathbf{p}}_i \cdot \hat{\mathbf{p}}_j))}{2} \tag{B.27}$$

With this angle, the maximum theoretical transparency for a grid with spherical apertures and a bridge thickness $t_{\text{bridge}} = 0$ can be calculated. Under these conditions the area of the spherical cap A_{cap}, which defines a single aperture, becomes:

$$A_{\text{cap}} := 2\pi(1 - \cos(\theta_{\text{min}})) \tag{B.28}$$

The maximum theoretical grid transparency $\eta_{\text{circular,max}}$ with equal sized apertures of half angle θ_{min} is then defined as:

$$\eta_{\text{circular,max}} = \frac{N \cdot A_{\text{cap}}}{A_{\text{sphere}}} = \frac{1}{2} N(1 - \cos(\theta_{\text{min}})) \tag{B.29}$$

If a finite bridge thickness t_{bridge} is assumed at the point of the smallest distance between two apertures, the reduced surface area of the caps $A_{\text{cap, red}}$ based on the half-angle α_{bridge} (see Eq. 5) becomes:

$$A_{\text{cap, red}} = 2\pi(1 - \cos(\theta_{\text{min}} - \alpha_{\text{bridge}})) \tag{B.30}$$

The transparency η_{circular} is then calculated analogously to Eq. B.29:

$$\eta_{\text{circular}} = \frac{1}{2} N(1 - \cos(\theta_{\text{min}} - \alpha_{\text{bridge}})) \tag{B.31}$$

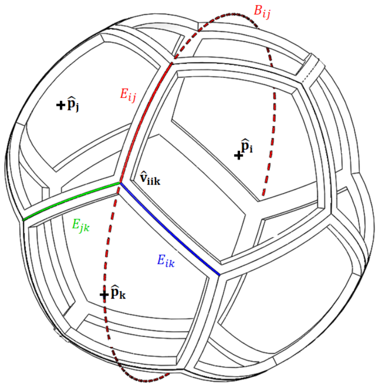

Figure B.3. Spherical Voronoi structure with sites $\hat{\mathbf{p}}_i, \hat{\mathbf{p}}_j$ and $\hat{\mathbf{p}}_k$ superimposed onto regular shaped grid structure with polygonal shaped apertures based on a dodecahedron ($N = 12$).

B.3.2 Polygonal Apertures

The calculation of the transparency for polygonal apertures is less trivial compared with the spherical apertures. Assuming a constant size of the structures between the apertures, the structures can be described by so-called spherical Voronoi diagrams. In the following paragraph, first, the basics of spherical Voronoi diagrams are reviewed and how the concept applies to regular-shaped grid geometries.

In its simplest 2D form a Voronoi diagram describes the partition of a plane into regions based on points (usually called sites) located on the plane in which each region has the shortest Euclidean distance to one site. In the case of the spherical Voronoi diagram the partition does not happen on a plane but on the surface of a sphere and the sites are defined by the center points of the equally distributed grid apertures. For an in-depth description and the complex calculation process of spherical Voronoi diagrams, see[7–11]. The spherical Voronoi diagrams were computed with the scipy.spatial.SphericalVoronoi class of the Python SciPy library[12]. In the following a brief description of spherical Voronoi diagrams is presented and how individual elements are related to the definition of regular grids with polygonal apertures.

The Voronoi diagrams are constructed from three main elements, which are displayed in Fig. B.3: 1. Bisectors, which form the base for 2. Voronoi edges (which represent the center line of the bridge segments of the regular grids with polygonal apertures) and 3. Voronoi vertices (which represent the joints of the polygonal grids).

The following paragraph mainly follows the description of[13]. The bisector B_{ij} is comprised of the set of points that are equidistant to the sites $\hat{\mathbf{p}}_i$ and $\hat{\mathbf{p}}_j$:

$$B_{ij} = \{\hat{\mathbf{r}} \in S^2 : d(\hat{\mathbf{r}}, \hat{\mathbf{p}}_i) = d(\hat{\mathbf{r}}, \hat{\mathbf{p}}_j)\} \tag{B.32}$$

Therefore, the bisector describes a great circle. To describe the Voronoi edge E_{ij} between two adjacent Voronoi cells with the boundaries $\partial V(\hat{\mathbf{p}}_i)$ and $\partial V(\hat{\mathbf{p}}_j)$ of their respective convex regions the intersection has to be calculated:

$$E_{ij} = \partial V(\hat{\mathbf{p}}_i) \cap B_{ij} = \partial V(\hat{\mathbf{p}}_j) \cap B_{ij} \tag{B.33}$$

The vertices $\hat{\mathbf{v}}_{i,j....k}$ shared by multiple apertures can be described as:

$$\hat{\mathbf{v}}_{i,j....k} = \{\hat{\mathbf{r}} \in \partial V(\hat{\mathbf{p}}_i) : d(\hat{\mathbf{r}}, \hat{\mathbf{p}}_i) = d(\hat{\mathbf{r}}, \hat{\mathbf{p}}_j) = ... = d(\hat{\mathbf{r}}, \hat{\mathbf{p}}_k)\} \tag{B.34}$$

In the context of this analysis a Voronoi edge describes the center-arc of a bridge segment and a Voronoi vertex represents the center-point where multiple segments meet. The calculation of the grid transparency is more complicated for grids with polygonal apertures compared to grids with circular apertures. The surface area of an individual spherical Voronoi cell can be calculated by dividing the cell into spherical triangles. One way to calculate the area of a spherical triangle A_{triangle} is to use Girard's theorem:

$$A_{\text{triangle}} = \alpha + \beta + \gamma - \pi \tag{B.35}$$

with α, β, γ being the angles of the spherical triangle. However, a less well-known formula presented by Eriksson[14] was found to be more suitable:

$$\tan\left(\frac{A_{\text{triangle}}}{2}\right) = \frac{|\mathbf{a} \cdot (\mathbf{b} \times \mathbf{c})|}{1 + \mathbf{a} \cdot \mathbf{b} + \mathbf{b} \cdot \mathbf{c} + \mathbf{a} \cdot \mathbf{c}} \tag{B.36}$$

where the area A_{triangle} is formed by the vectors $\mathbf{a}, \mathbf{b}$ and $\mathbf{c}$ which represent the vector of one site and two corresponding Voronoi vertices that form (one part) of the spherical triangle of a Voronoi cell. To account for the finite thickness of the bridges between the apertures, a set of "reduced" vertices $\hat{\mathbf{v}}_{i,\text{red}}$ needs to be created (three $\hat{\mathbf{v}}_{i,\text{red}}$ per $\hat{\mathbf{v}}_i$). The vertices are rotated by the angle α_{bridge} around the normal vector between the vertex $\hat{\mathbf{v}}_i$ and the corresponding center point $\hat{\mathbf{p}}_i$. This rotation can be calculated with the Euler-Rodrigues rotation formula[15]. By defining the rotation vector $\mathbf{k}_{\text{rot}}$:

$$\mathbf{k}_{\text{rot},i} = \frac{\hat{\mathbf{v}}_i \times \hat{\mathbf{p}}_i}{||\hat{\mathbf{v}}_i \times \hat{\mathbf{p}}_i||} \tag{B.37}$$

The reduced vertices $\hat{\mathbf{v}}_{i,\text{red}}$ can then be calculated by:

$$\hat{\mathbf{v}}_{i,\text{red}} = \hat{\mathbf{v}}_i \cos(\alpha_{\text{bridge}}) + (\mathbf{k}_{\text{rot},i} \times \hat{\mathbf{v}}_i) \sin(\alpha_{\text{bridge}}) + \mathbf{k}_{\text{rot},i}(\mathbf{k}_{\text{rot},i} \cdot \hat{\mathbf{v}}_i)(1 - \cos(\alpha_{\text{bridge}})) \tag{B.38}$$

For quadrilateral, pentagonal and hexagonal-shaped apertures and apertures with more edges, the n_i-sided surface areas can be calculated by dividing them into n_i spherical triangles (the three vertices defining the triangle are the center-point $\hat{\mathbf{p}}_i$ and the two vertices of one of the edges of the aperture), calculate the individual surface areas $A_{\text{triangle},h}$ and then simply compute their total sum $A_{\text{polygonal},i}$:

$$A_{\text{polygonal},i} = \sum_{h=1}^{n_i} A_{\text{triangle},h} \tag{B.39}$$

The transparency of the grid with polygonal apertures $\eta_{\text{polygonal}}$ is calculated by dividing the total sum of the individual areas of the polygonal apertures $A_{\text{polygonal},i}$ by the total area of the underlying sphere.

$$\eta_{\text{polygonal}} = \frac{\sum_{i=1}^{N} A_{\text{polygonal,i}}}{4\pi R_{\text{grid}}^2} \tag{B.40}$$

An important question is how many edges the grid apertures will have in order to estimate how regularly the grids will be shaped. Euler's theorem for convex polyhedra with f faces, e edges and v vertices states[16]:

$$f - e + v = 2 \tag{B.41}$$

If the number of faces f is substituted with the equal number of apertures N and it is taken into account that for each n-sided aperture the bridge (edge) is shared by two apertures and every corner (vertices) is shared by three other apertures, the following equation with N_n being the number of n-sided apertures is obtained:

$$\sum_n (N_n n/3 - N_n n/2 + N_n) = 2 \tag{B.42}$$

This equation can be simplified to:

$$\sum_n (6 - n) N_n = 12 \tag{B.43}$$

Therefore, the average number of edges per aperture becomes:

$$\frac{\sum_n n N_n}{N} = 6 - \frac{12}{N} \tag{B.44}$$

This equation shows that the average number of apertures is less than 6. In the case of 120 apertures, the average number of edges per aperture becomes 5.9. For example, the equation predicts correctly the average number of edges per aperture for the Buckyball geometry (5.625 for 20 hexagons and 12 pentagons).

B.4 Implementation of Code

The code was implemented into Python 3. By making extensive use of the NumPy-library[17] (especially the aggregations and broadcasting capability to speed up the calculation) and the SciPy's scipy.spatial.SphericalVoronoi class[12] the code was kept simple.

The following parameters were used for the optimization study:

- Number of apertures: $N = 6$ to 120
- Number of iterations by optimization by the potential gradient (see Eq. B.21 and B.22): $n_{\text{pot,max}} = 1$ (It was found that the optimization of the dot-product delivered the necessary and precise convergence without prior optimization by the energy gradient.)
- s-Energy (see Eq. 11): $s = 2$
- Weighting factor of potential gradient: $\varepsilon_1 = 10^{-3}$ (as suggested by Gautam and Vaintrob[4].)
- Number of iterations by optimization by dot product (see Eq. B.23, B.24 and B.25): $n_{\text{dot,max}} = 10000$
- Weighting factor of dot-product (see Eq. B.26): $\varepsilon_2(n_{\text{dot}}) = \exp(-n_{\text{dot}}^{3/4}/500)$ (as suggested by Gautam and Vaintrob[4].)
- Total iterations for each configuration: 10. The solution with the highest transparency η was selected.
- Two datasets for each number of aperture were calculated: One set with antipodal symmetry and one without the antipodal constraint (only for comparison).

IECF grids require an antipodal symmetry for their apertures to allow for circulating ion beams through the core region (see RQ2 in Section 2.2 and Eq. 4. Additionally to the antipodally constrained configurations, a second set of antipodally unconstrained configurations was calculated. These configurations have a higher transparency (see Fig. 17). The results for even (unconstrained) and antipodal constrained configuration (the basis for regular grids, see Eq. 4) were tested against data provided by Conway et al.[18] ($N = 6$ to 110, only even values) for the antipodal constrained configuration and Sloane et al.[19] ($N = 6$ to 120) for the unconstrained symmetry. The results are shown in Fig. B.4. It can be seen that the relative difference is usually lower than 0.2 % for both configurations. With maximum differences only slightly surpassing 1 percent in one case ($N = 110$) the precision was deemed acceptable for the use of analyzing the geometries for spherical IECF cathodes.

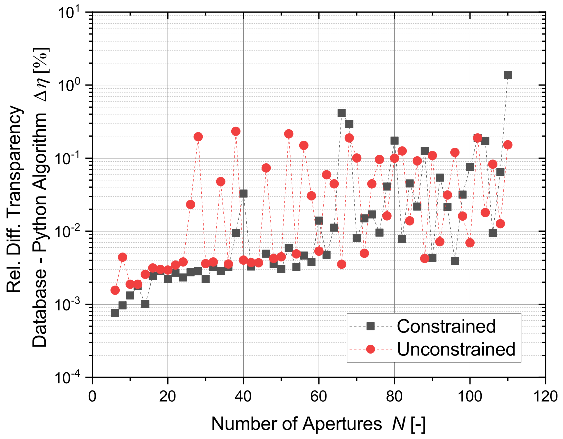

Figure B.4. Relative differences between the distributions calculated with algorithm described above and data present by Sloane et al.[18,19].

The maximum number of apertures of $N = 120$ was chosen based on the following aspects:

- The maximum number of apertures for symmetric grids seems to be 120 (see Chapter 6).
- There is only reliable data for comparison up to $N = 110$ (see database[19]).
- Computational complexity (the algorithm as well as the geometry generation in Salome Meca require significant computational time for higher N)

References

1. Tammes, P. M. L. On the origin of number and arrangement of the places of exit on the surface of pollen-grains. *Recueil des travaux botaniques néerlandais* **27**, 1–84 (1930).
2. Thomson, J. XXIV. On the structure of the atom: an investigation of the stability and periods of oscillation of a number of corpuscles arranged at equal intervals around the circumference of a circle; with application of the results to the theory of atomic structure. *The London, Edinburgh, Dublin Philos. Mag. J. Sci.* **7**, 237–265, DOI: 10.1080/14786440409463107 (1904).
3. Conway, J. H. & Sloane, N. J. A. *Sphere Packings and Kissing Numbers*, vol. 290 (Springer New York, 1999).

4. Gautam, S. & Vaintrob, D. A Novel Approach to the Spherical Codes Problem.

5. Saff, E. B. & Kuijlaars, A. B. J. Distributing many points on a sphere. *The Math. Intell.* **19**, 5–11, DOI: 10.1007/BF03024331 (1997).

6. Katanforoush, A. & Shahshahani, M. Distributing Points on the Sphere, I. *Exp. Math.* **12**, 199–209, DOI: 10.1080/10586458.2003.10504492 (2003).

7. Augenbaum, J. M. & Peskin, C. S. On the construction of the Voronoi mesh on a sphere. *J. Comput. Phys.* **59**, 177–192, DOI: 10.1016/0021-9991(85)90140-8 (1985).

8. Renka, R. J. Algorithm 772. *ACM Transactions on Math. Softw.* **23**, 416–434, DOI: 10.1145/275323.275329 (1997).

9. Na, H.-S., Lee, C.-N. & Cheong, O. Voronoi diagrams on the sphere. *Comput. Geom.* **23**, 183–194, DOI: 10.1016/S0925-7721(02)00077-9 (2002).

10. Larrea, M., Urribarri, D., Martig, S. & Castro, S. M. Spherical Layout Implementation using Centroidal Voronoi Tessellations. *J. Comput.* **1**, 81–86, DOI: 10.48550/arXiv.0912.3974 (2009).

11. Dinis, J. & Mamede, M. Sweeping the Sphere. In *2010 International Symposium on Voronoi Diagrams in Science and Engineering*, 151–160, DOI: 10.1109/ISVD.2010.32 (IEEE, 2010).

12. Virtanen, P. *et al.* SciPy 1.0: fundamental algorithms for scientific computing in Python. *Nat. Methods* **17**, 261–272, DOI: 10.1038/s41592-019-0686-2 (2020).

13. Zheng, X. & Ennis, R. A Plane Sweep Algorithm for the Voronoi Tessellation of the Sphere. *Electron. crystal Commun. (e-LC)* 1–13 (2011).

14. Eriksson, F. On the Measure of Solid Angles. *Math. Mag.* **63**, 184, DOI: 10.2307/2691141 (1990).

15. Cheng, H. & Gupta, K. C. An Historical Note on Finite Rotations. *J. Appl. Mech.* **56**, 139–145, DOI: 10.1115/1.3176034 (1989).

16. Euler, L. Elementa doctrinae solidorum. *Novi Commentarii academiae scientiarum Petropolitanae* **4**, 109–140 (1758).

17. Harris, C. R. *et al.* Array programming with NumPy. *Nature* **585**, 357–362, DOI: 10.1038/s41586-020-2649-2 (2020).

18. Conway, J. H., Hardin, R. H. & Sloane, N. J. A. Packing Lines, Planes, etc.: Packings in Grassmannian Spaces. *Exp. Math.* **5**, 139–159, DOI: 10.1080/10586458.1996.10504585 (1996).

19. Sloane, N. J. A. Spherical Codes Nice arrangements of points on a sphere in various dimensions.
